# GWAS-significant loci and severe COVID-19: analysis of associations, link with thromboinflammation syndrome, gene-gene, and gene-environmental interactions

**DOI:** 10.3389/fgene.2024.1434681

**Published:** 2024-08-08

**Authors:** Alexey Valerevich Loktionov, Ksenia Andreevna Kobzeva, Andrey Romanovich Karpenko, Vera Alexeevna Sergeeva, Yuriy Lvovich Orlov, Olga Yurievna Bushueva

**Affiliations:** ^1^ Department of Anesthesia and Critical Care, Institute of Continuing Education, Kursk State Medical University, Kursk, Russia; ^2^ Laboratory of Genomic Research, Research Institute for Genetic and Molecular Epidemiology, Kursk State Medical University, Kursk, Russia; ^3^ Institute of Biodesign and Complex Systems Modeling, Sechenov First Moscow State Medical University (Sechenov University), Moscow, Russia; ^4^ Department of Biology, Medical Genetics and Ecology, Kursk State Medical University, Kursk, Russia

**Keywords:** chronic diseases, genotyping, COVID-19, GWAS, thromboinflammation syndrome, rs17713054, rs17078346, rs12610495

## Abstract

**Objective:**

The aim of this study was to replicate associations of GWAS-significant loci with severe COVID-19 in the population of Central Russia, to investigate associations of the SNPs with thromboinflammation parameters, to analyze gene-gene and gene-environmental interactions.

**Materials and Methods:**

DNA samples from 798 unrelated Caucasian subjects from Central Russia (199 hospitalized COVID-19 patients and 599 controls with a mild or asymptomatic course of COVID-19) were genotyped using probe-based polymerase chain reaction for 10 GWAS-significant SNPs: rs143334143 *CCHCR1*, rs111837807 *CCHCR1*, rs17078346 *SLC6A20-LLZTFL1*, rs17713054 *SLC6A20-LLZTFL1*, rs7949972 *ELF5*, rs61882275 *ELF5*, rs12585036 *ATP11A*, rs67579710 *THBS3, THBS3-AS1*, rs12610495 *DPP9*, rs9636867 *IFNAR2*.

**Results:**

SNP rs17713054 *SLC6A20-LZTFL1* was associated with increased risk of severe COVID-19 in the entire group (risk allele A, OR = 1.78, 95% CI = 1.22–2.6, *p* = 0.003), obese individuals (OR = 2.31, 95% CI = 1.52–3.5, *p* = 0.0002, (*p*_bonf_ = 0.0004)), patients with low fruit and vegetable intake (OR = 1.72, 95% CI = 1.15–2.58, *p* = 0.01, (*p*_bonf_ = 0.02)), low physical activity (OR = 1.93, 95% CI = 1.26–2.94, *p* = 0.0035, (*p*_bonf_ = 0.007)), and nonsmokers (OR = 1.65, 95% CI = 1.11–2.46, *p* = 0.02). This SNP correlated with increased BMI (*p* = 0.006) and worsened thrombodynamic parameters (maximum optical density of the formed clot, D (*p* = 0.02), delayed appearance of spontaneous clots, Tsp (*p* = 0.02), clot size 30 min after coagulation activation, CS (*p* = 0.036)). SNP rs17078346 *SLC6A20-LZTFL1* was linked with increased BMI (*p* = 0.01) and severe COVID-19 in obese individuals (risk allele C, OR = 1.72, 95% CI = 1.15–2.58, *p* = 0.01, (*p*_bonf_ = 0.02)). SNP rs12610495 *DPP9* was associated with increased BMI (*p* = 0.01), severe COVID-19 in obese patients (risk allele G, OR = 1.48, 95% CI = 1.09–2.01, *p* = 0.01, (*p*_bonf_ = 0.02)), and worsened thrombodynamic parameters (time to the start of clot growth, Tlag (*p* = 0.01)). For rs7949972 *ELF5*, a protective effect against severe COVID-19 was observed in non-obese patients (effect allele T, OR = 0.67, 95% CI = 0.47–0.95, *p* = 0.02, (*p*_bonf_ = 0.04)), improving thrombodynamic parameters (CS (*p* = 0.02), stationary spatial clot growth rates, Vst (*p* = 0.02)). Finally, rs12585036 *ATP11A* exhibited a protective effect against severe COVID-19 in males (protective allele A, OR = 0.51, 95% CI = 0.32–0.83, *p* = 0.004). SNPs rs67579710 *THBS3*, *THBS3*-*AS1*, rs17713054 *SLC6A20-LZTFL1*, rs7949972 *ELF5,* rs9636867 *IFNAR2*—were involved in two or more of the most significant G×G interactions (*p*_perm_ ≤ 0.01). The pairwise combination rs67579710 *THBS3*, *THBS3*-*AS1* × rs17713054 *SLC6A20-LZTFL1* was a priority in determining susceptibility to severe COVID-19 (it was included in four of the top five most significant SNP-SNP interaction models).

**Conclusion:**

Overall, this study represents a comprehensive molecular-genetic and bioinformatics analysis of the involvement of GWAS-significant loci in the molecular mechanisms of severe COVID-19, gene-gene and gene-environmental interactions, and provides evidence of their relationship with thromboinflammation parameters in patients hospitalized in intensive care units.

## 1 Introduction

The emergence of coronavirus disease 2019 (COVID-19) at the close of 2019 brought forth an array of symptoms and outcomes stemming from Severe Acute Respiratory Syndrome Coronavirus 2 (SARS-CoV-2). Globally, the case fatality rate of COVID-19 ranges from 1% to 17%. Various factors, like the size of the population tested, demographic characteristics, ethnicity, the effectiveness of healthcare systems, and virus variants can affect the rates of mortality from COVID-19 (https://coronavirus.jhu.edu/data/mortality (accessed 18 March 2024)). However, the most common cause of death from COVID-19 is severe disease, manifested by immune dysregulation and the onset of a cytokine storm (CS) (Kim et al., 2021), characterized by a rapid surge in proinflammatory cytokines and other markers of inflammation. This hyperinflammation leads to coagulopathies, oxidative stress, organ failure, and ultimately, mortality (Silva et al., 2023). Hypercoagulation and micro-clot formation are critical factors in the molecular pathogenesis of COVID-19, contributing significantly to its complications and adverse outcomes (Pretorius et al., 2020). Furthermore, we are becoming increasingly aware of COVID-19 long-term consequences on various organ systems, including the pulmonary, cardiovascular, hematologic, renal, central nervous system, gastrointestinal, and psychosocial manifestations (Joshee et al., 2022; Ma et al., 2022). This growing comprehension underscores the imperative to delve deeper into the understanding of COVID-19.

Understanding why some individuals experience asymptomatic or mild courses while others face intensive care unit (ICU) admissions with severe organ failure and mortality remains a critical challenge and the subject of much research worldwide (Carvalho et al., 2023; Collins et al., 2023).

To date, it is known that lifestyle factors such as fruit and vegetable consumption and physical activity significantly influence the severity of COVID-19 (Yedjou et al., 2021; Tadbir Vajargah et al., 2022; Tavakol et al., 2023). However, host genetic factors play no less a significant role, as evidenced by findings from molecular-genetic studies. Genes such as *SLC6A20*, *LZTFL1*, *IFNAR2*, *DPP9*, *CCHCR1*, *ELF5*, *ATP11A* and *THBS3* have been identified as potentially contributing to severe COVID-19 and hospitalization in genome-wide association studies (Severe Covid-19 GWAS Group et al., 2020; Lee et al., 2021; Horowitz et al., 2022; Kousathanas et al., 2022; Pairo-Castineira et al., 2021). Many of the genetic variants identified by GWAS have been replicated in different populations around the world, demonstrating their high predictive value for the risk of severe COVID-19 (Rescenko et al., 2021; Garg et al., 2024).

Despite the wealth of genetic data, there is a significant lack of research worldwide on the relationship between genetic variants and the severity of thromboinflammatory syndrome in COVID-19 patients, as well as intergenic interactions, interactions between genetic variants and environmental factors that could either mitigate or exacerbate the impact of genetic variants on the severity of the disease.

Therefore, the aim of this pilot study was to i) investigate the association between common single nucleotide polymorphisms identified by GWAS and the risk of severe COVID-19 in a Russian population; ii) investigate the most significant gene-gene interactions associated with severe COVID-19; iii) evaluate the joint influence of polymorphisms and environmental risk factors on disease susceptibility; and iv) find out how COVID-19 GWAS loci influence the features of the clinical manifestations of the disease, including thrombodynamic parameters.

## 2 Materials and methods

### 2.1 Study design

**FIGURE 1:**
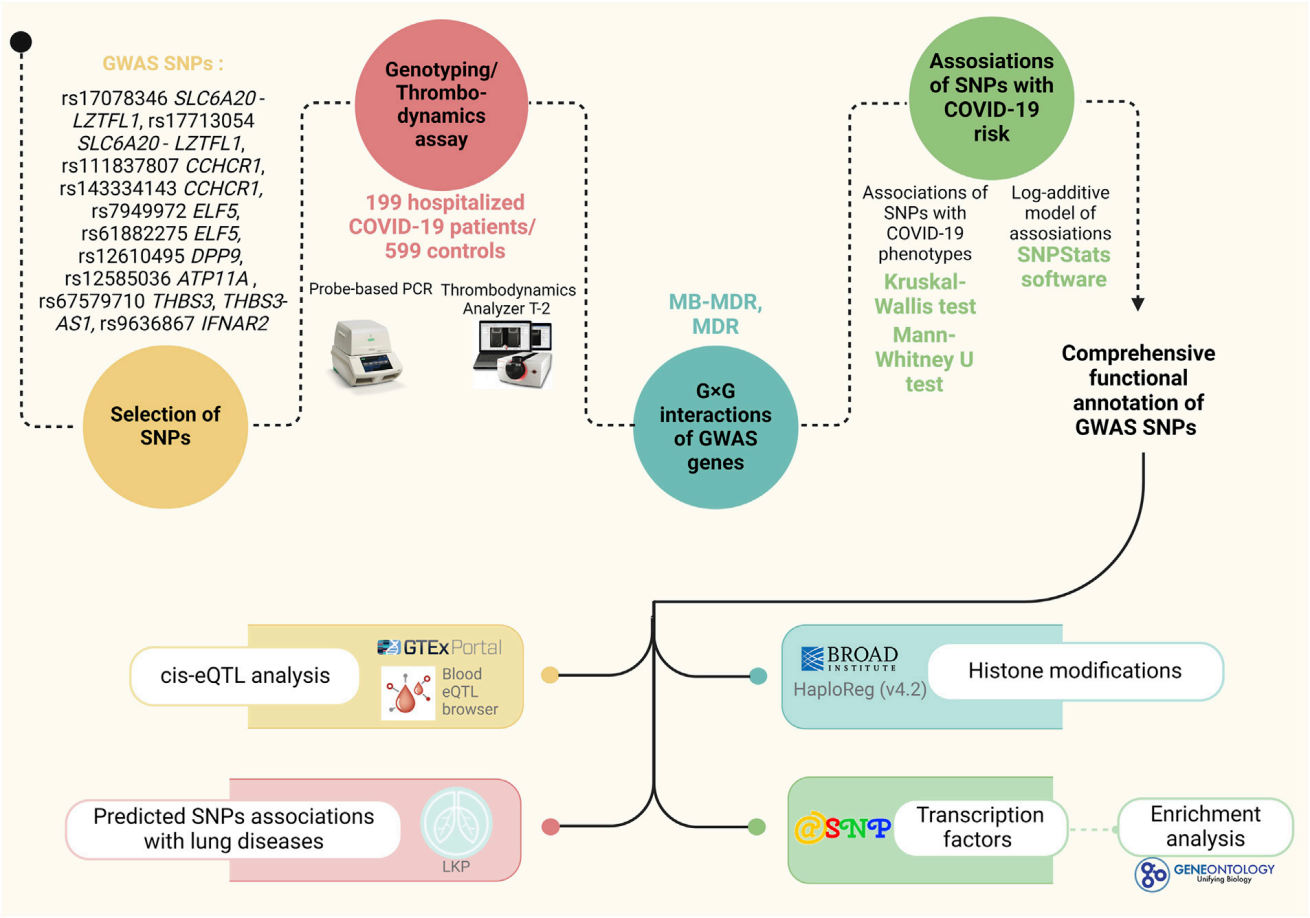
Materials and methods of the study.

The study’s fundamental structure, along with the materials and tools employed, are outlined in Figure 1.

### 2.2 Study participants

The study included 798 unrelated individuals from Central Russia, comprising 199 hospitalized COVID-19 patients and 599 patients of the control group. The Ethical Review Committee of Kursk State Medical University approved the study protocol (protocol №1 from 11 January 2022), and all participants provided written informed consent. The patients were enrolled in the study during the COVID-19 pandemic from 2020 to 2022 at the intensive care units (ICU) of Kursk Regional Hospital №6 and Kursk Regional Tuberculosis Dispensary. All patients had a PCR-confirmed diagnosis of COVID-19. The control group consisted of healthy volunteers from Biobank of Research Institute for Genetic and Molecular Epidemiology (Bushueva O. et al., 2015; OYu et al., 2015) who had mild or asymptomatic COVID-19 and did not need ICU admission (Bushueva, 2020; Kobzeva et al., 2022; Belykh et al., 2023). Supplementary Table S1 provides the baseline and clinical characteristics of the study cohort.

In accordance with WHO guidelines (Amine et al., 2003), low fruit and vegetable consumption was defined as consuming less than 400 g per day. Adequate consumption of fresh vegetables and fruits was defined as consuming 400 g or more, equivalent to 3-4 servings per day, excluding starchy tubers like potatoes. Insufficient physical activity was characterized by engaging in less than 180 min per week of moderate to vigorous physical activities. This encompassed various forms of exercise, including leisure activities such as walking and running as well as fitness club exercises like treadmill running, aerobics, or resistance training. Obesity is assessed using the Body Mass Index (BMI), a measurement based on a person’s height and weight. A BMI of 30 or higher is generally considered indicative of obesity.

### 2.3 Selection of genes and polymorphisms

For this study, we selected SNPs from the largest GWAS meta-analysis of severe COVID-19 (top 20 SNPs with *p*-level of significance of ≤1 × 10^−20^) (Pairo-Castineira et al., 2023). Then, SNPs with a minor allele frequency <0.05 were excluded from the analysis, as well as loci for which was unable to design probes for TaqMan-based-PCR (low CG composition, presence of GC clamps, runs of identical nucleotides). In total, 10 SNPs were included in the genotyping: rs143334143 *CCHCR1* (chr6:31153649 (GRCh38)), rs111837807 *CCHCR1* (chr6:31153455 (GRCh38)), rs17078346 *SLC6A20*-*LZTFL1*(chr3:45804256 (GRCh38)), rs17713054 *SLC6A20*-*LZTFL1* (chr3:45818159 (GRCh38)), rs7949972 *ELF5* (chr11:34480495 (GRCh38)), rs61882275 *ELF5* (chr11:34482745 (GRCh38)), rs12585036 *ATP11A* (chr13:112881427 (GRCh38)), rs67579710 *THBS3*, *THBS3*-*AS1* (chr1:155203736 (GRCh38)), rs12610495 *DPP9* (chr19:4717660 (GRCh38)), rs9636867 *IFNAR2* (chr21:33639 (GRCh38)).

### 2.4 Genetic analysis

The Laboratory of Genomic Research at the Research Institute for Genetic and Molecular Epidemiology of Kursk State Medical University (Kursk, Russia) performed genotyping. Up to 5 mL of venous blood from each participant was collected from a cubital vein, put into EDTA-coated tubes, and kept at −20 C until it was processed. Defrosted blood samples were used to extract genomic DNA using the standard methods of phenol/chloroform extraction and ethanol precipitation. The purity, quality, and concentration of the extracted DNA samples were assessed using a NanoDrop spectrophotometer (Thermo Fisher Scientific, Waltham, MA, United States).

Genotyping of the SNPs was performed using allele-specific probe-based polymerase chain reaction (PCR) according to the protocols designed in the Laboratory of Genomic Research at the Research Institute for Genetic and Molecular Epidemiology of Kursk State Medical University. The Primer3 software was used for primer design (Koressaar and Remm, 2007). A real-time PCR procedure was performed in a 25 µL reaction solution containing 1.5 units of Hot Start Taq DNA polymerase (Biolabmix, Novosibirsk, Russia), approximately 10 ng of DNA, and the following concentrations of reagents: 0.25 μM of each primer; 0.1 μM of each probe; 250 μM of each dNTP; 3 mM MgCl_2_ for rs7949972, 3.5 mM MgCl_2_ for rs61882275, 2 mM MgCl_2_ for rs12610495, and 2.5 mM MgCl_2_ for the remining SNPs; 1xPCR buffer (67 mM Tris-HCl, pH 8.8, 16.6 mM (NH_4_)_2_SO_4_, 0.01% Tween-20). The PCR procedure comprised an initial denaturation for 10 min at 95°C, followed by 39 cycles of 92 °C for 30 s and 57 °C, 59 °C, 60 °C, 61 °C, 62 °C, 63 °C, 65 °C, 66 °C for 1 min (for rs12610495 *DPP9*, rs17078346 *SLC6A20*-*LZTFL1*, rs17713054 *SLC6A20*-*LZTFL1*, rs111837807 *CCHCR1*, rs9636867 *IFNAR2*, rs143334143 *CCHCR1* and rs7949972 *ELF5*, rs12585036 *ATP11A* and rs61882275 *ELF5*, rs67579710 *THBS3*, *THBS3*-*AS1*, respectively). 10% of the DNA samples were genotyped twice, blinded to the case-control status, in order to assure quality control. Over 99% of the data were concordant. Due to the Hardy-Weinberg equilibrium deviation in the control group for SNP rs12610495 *DPP9*, all locus samples underwent re-genotyping. The results were entirely consistent (100%) with the initial genotypes.

### 2.5 Thrombodynamics analysis

The analysis utilized venous blood samples obtained from the peripheral veins of patients upon admission to the ICU, prior to the initiation of drug therapy or any other manipulations. Blood collection involved vacuum tubes containing sodium citrate 3.2%, with a maximum interval of 45 min between collection and centrifugation.

To isolate platelet-free plasma for the thrombodynamics test, a “soft” double centrifugation method was used: samples underwent initial centrifugation at 1,600 *g* for 15 min, followed by an additional 20 min at 1,600 g. Platelet-free plasma (120 µL) was used for the test within 3 h.

The thrombodynamics test was performed using the laboratory diagnostic system “Thrombodynamics Recorder TD-2". Blood plasma was introduced into specialized cuvettes, into which an “activator-insert” containing lipids and tissue factor protein was added. This factor initiated the clotting process, simulating damage to the blood vessel wall. Coagulation is initiated on the surface of an activator fixed in space and extends into a thin layer of non-stirred plasma. The growth of the fibrin clot was recorded by the device in sequential photography mode with a digital camera using the dark field method for 30 min.

Based on the obtained images, the Thrombodynamics Recorder TD-2 software calculated the quantitative parameters of the spatial dynamics of fibrin clot growth and spontaneous thrombus formation, including: time to the start of clot growth (Tlag), initial Vi) and stationary (Vst) spatial clot growth rates (the slopes of the clot size curve vs time for the segments of 2–6 min and 15–25 min from the clot growth start for Vi and V, respectively), the clot size at 30 min after coagulation activation (CS), the maximum optical density of the formed clot (D), characterizing its quality, and the time of appearance of spontaneous clots in the sample (Tsp). This latter characteristic has substantial clinical value because spontaneous clots (i.e., those that do not grow from the activator surface) may only be observed in cases of serious hypercoagulable states.

### 2.6 Statistical and bioinformatic analysis

The STATISTICA software (v13.3, United States) was utilized for statistical processing. The normality of the distribution for quantitative data was assessed using the Shapiro-Wilk’s test. Given that the majority of quantitative parameters exhibited deviations from normal distribution, they were presented as the median (Me) along with the first and third quartiles [Q1 and Q3]. The Kruskal–Wallis test was used to compare quantitative variables among three independent groups. Following that, groups were contrasted pairwise using the Mann–Whitney test. To compare quantitative variables among two independent groups, the Mann-Whitney test was also performed. For categorical variables, differences in statistical significance were evaluated using Pearson’s chi-squared test with Yates’s correction for continuity.

The compliance of genotype distributions with Hardy-Weinberg equilibrium was evaluated using Fisher’s exact test. The study groups’ genotype frequencies and their associations with disease risk were analyzed using the SNPStats software (https://www.snpstats.net/start.htm (accessed on 18 February 2024)). The additive model was considered for the genotype association analysis. Associations within the entire group of COVID-19 patients/controls were adjusted for age and gender. Given the potentially significant modifying influence of environmental risk factors on the association of genetic markers with disease (Bushueva et al., 2016; Polonikov et al., 2017), associations were analyzed based on the presence or absence of the risk factor. When information about the environmental risk factor was unavailable in the control group (for fruit/vegetable intake, physical activity levels), the patient group was compared to the overall control group. In such cases, the Bonferroni correction was applied to account for multiple comparisons.

The MB-MDR analysis tested two-, three-, and four-level genotype combinations (G×G) and genotype-environment combinations with the including of smoking as an environmental risk factor (G×E). Smoking was analyzed as an environmental risk factor in the analysis of G×E interactions (due to the high pathogenetic significance of this environmental factor in the development of severe COVID-19, as well as the lack of data about other environmental factors like physical activity levels and levels of fruit and vegetable intake in control group). Since SNPs located in the same genes are in linkage disequilibrium, and linkage groups included no more than two SNPs, one of the SNPs was included in the MB-MDR analysis. For each model, the empirical *p*-value (*p*_perm_) was estimated using a permutation test. Permutation testing was employed to improve the validity of the results obtained (Calle et al., 2010). Because the default call to MB-MDR is designed to simultaneously test all possible interactions of a given order, we used 1,000 permutations to obtain accurate p-values. Models with *p*_perm_ < 0.01 were considered as statistically significant. All calculations were adjusted for gender and age. Statistical analysis was carried out using the R software environment. Models (on average 3-4 models of each level) with the highest Wald statistics and the lowest *p*-level of significance were included in the study. Additionally, using the MB-MDR method, individual combinations of genotypes associated with the studied phenotypes were established (*p* < 0.05). Calculations were performed in the MB-MDR program for the R software environment (Version 3.6.3) (Ivanova, 2024).

Additionally, the most significant G×G and G×E models were analyzed using the MDR method (the analysis included genes that appeared in the 2 or more best models of 2-, 3- and 4-locus G×G models in the analysis of intergenic interactions/smoking and genes included in 2 or more best models of 2-, 3- and 4-locus G×E models in the analysis of gene-environment interactions with the including of smoking as an environmental risk factor). The analysis was implemented in the MDR program (v.3.0.2) (http://sourceforge.net/projects/mdr (accessed on 25 February 2024)). The MDR method was used to assess the mechanisms of interactions (synergy, antagonism, additive interactions (independent effects)) and the strength of interactions (the contribution of individual genes/environmental factors as the purpose of the study, to the entropy of a trait and the contribution of interactions, calculated as a percentage). The results of the MDR analysis were visualized as a graph.

We conducted a mediation analysis using the “statsmodels” package for Python to assess whether rs17713054, identified as a genetic risk factor in overall group in our study, influences SARS-CoV-2 directly or indirectly through other clinical conditions such as essential hypertension (EH), coronary artery disease (CAD), cerebrovascular accident (CVA) in anamnesis, chronic obstructive pulmonary disease (COPD), and diabetes mellitus type 2 (T2D).

The functional effects of SNPs were examined using bioinformatics resources, the methodologies and functionalities of which were comprehensively described in our prior research (Kobzeva et al., 2023; Shilenok et al., 2023; Stetskaya et al., 2024):

• The bioinformatic tool GTExportal (http://www.gtexportal.org/ (accessed on 28 February 2024)) was used to analyze the link of SNPs with expression quantitative trait loci (eQTLs) in lungs, whole blood, blood vessels, and adipose tissue (Consortium, 2020).
• For additional examination of binding SNPs to expression quantitative trait loci (eQTL) in peripheral blood, the eQTLGen resource available at https://www.eqtlgen.org/ (accessed on 28 February 2024) was employed (Võsa et al., 2018).
• HaploReg (v4.2), a bioinformatics tool available at https://pubs.broadinstitute.org/mammals/haploreg/haploreg.php (accessed on 28 February 2024), was utilized to assess the associations between GWAS SNPs and specific histone modifications marking promoters and enhancers. These modifications included acetylation of lysine residues at positions 27 and 9 of the histone H3 protein, as well as mono-methylation at position 4 (H3K4me1) and tri-methylation at position 4 (H3K4me3) of the histone H3 protein. Additionally, the tool was applied to investigate the positioning of SNPs in DNase hypersensitive regions (Ward and Kellis, 2012).
• The atSNP Function Prediction online tool (http://atsnp.biostat.wisc.edu/search (accessed on 29 February 2024)) was used to evaluate the impact of SNPs on the gene affinity to transcription factors (TFs) depending on the carriage of the reference/alternative alleles (Shin et al., 2019). TFs were included based on the degree of influence of SNPs on the interaction of TFs with DNA calculated on the basis of a positional weight matrix.
• Using the Gene Ontology online tool (http://geneontology.org/ (accessed on 29 February 2024)), it was feasible to analyze the joint involvement of TFs linked to the reference/SNP alleles in overrepresented biological processes directly related to the pathogenesis of severe COVID-19 (Consortium, 2019). Biological functions controlled by transcription factors associated with SNPs were used as functional groups.
• The Lung Disease Knowledge Portal (LKP) (https://cd.hugeamp.org/ (accessed on 29 February 2024)), which combines and analyzes the results of genetic associations of the largest consortiums for the study of lung diseases, was used for bioinformatics analysis of associations of SNPs with COVID-19 and intermediate phenotypes (such as FEV1, FEV1 to FVC ratio, etc.).

## 3 Results

### 3.1 Genetic correlates between GWAS- significant loci and the risk of severe COVID-19

The genotype frequencies of SNPs within the study cohorts are detailed in Supplementary Table S2. Because associations of genetic markers with disease can lead to deviations from equilibrium, we relied on the results of Hardy-Weinberg equilibrium analysis in the control group. Within the control group, all studied SNPs exhibited genotype frequencies consistent with Hardy-Weinberg equilibrium (*p* > 0.05), except for rs12610495 *DPP9* (Supplementary Table S2). However, due to the fact that repeated genotyping of rs12610495 showed 100% reproducibility of the primary results, this SNP was included in the statistical analysis.

The analysis of the entire group (Table 1) revealed an association between rs17713054 *SLC6A20*-*LZTFL1* and the increased risk of severe COVID-19 course, regardless of sex and age: risk allele A, OR = 1.78, 95% CI = 1.22–2.6, *p* = 0.003. Sex-stratified analysis (Supplementary Table S3) showed that rs17713054 *SLC6A20*-*LZTFL1* elevates the risk of severe COVID-19 both in males (OR = 1.91, 95% CI = 1.12–3.26, *p* = 0.02) and females (OR = 1.63, 95% CI = 1.03–2.58, *p* = 0.04); additionally, we found that rs12585036 *ATP11A* lowers the risk of severe COVID-19 in males (protective allele T; OR = 0.51, 95% CI = 0.32–0.83, *p* = 0.004).

**TABLE 1:** Results of the analysis of associations between GWAS SNPs and severe COVID-19 risk in the entire group.

Genetic variant	Effect allele	Other allele	N	OR [95% CI]^1^	*p* ^2^
rs143334143 *CCHCR1*	A	G	752	1.07 [0.72–1.59]	0.74
rs111837807 *CCHCR1*	C	T	751	0.98 [0.64–1.50]	0.94
rs17713054 *SLC6A20-LZTFL1*	A	G	753	**1.78 [1.22–2.60]**	**0.003**
rs17078346 *SLC6A20-LZTFL1*	C	A	754	1.41 [0.99–2.02]	0.059
rs12585036 *ATP11A*	T	C	749	0.87 [0.65–1.18]	0.37
rs12610495 *DPP9*	G	A	749	1.03 [0.79–1.34]	0.82
rs7949972 *ELF5*	T	C	743	0.92 [0.71–1.21]	0.56
rs61882275 *ELF5*	A	G	751	1.17 [0.91–1.51]	0.21
rs67579710 *THBS3*, *THBS3*-*AS1*	A	G	749	0.65 [0.41–1.05]	0.072
rs9636867 *IFNAR2*	G	A	751	0.85 [0.65–1.11]	0.24

All calculations were performed relative to the minor alleles (Effect allele) with adjustment for sex, age; 1 - odds ratio and 95% confidence interval; 2– *p*-value; statistically significant differences are marked in bold.

Mediation analysis revealed that the indirect effect of rs17713054 through T2D, CVA, and EH was insignificant, accounting for 0%, 2.79%, and 5.48% respectively, in the sequential analysis of these conditions. Adding these variables to the logistic regression model did not render the influence of rs17713054 on SARS-CoV-2 statistically insignificant.

Conversely, adding COPD or CAD to the model rendered the influence of rs17713054 on SARS-CoV-2 insignificant (with the model including COPD showing a weaker effect and the significance level remaining within the statistical trend, *p* < 0.1). Mediation analysis for these variables showed that the contribution of rs17713054 to SARS-CoV-2, mediated through COPD and CAD, was 18.57% and 71.54% respectively. This suggests that the influence of rs17713054 on SARS-CoV-2 is likely mediated through other clinical conditions, primarily through CAD (Supplementary Table S4).

### 3.2 Gene-gene interactions associated with severe COVID-19

**FIGURE 2:**
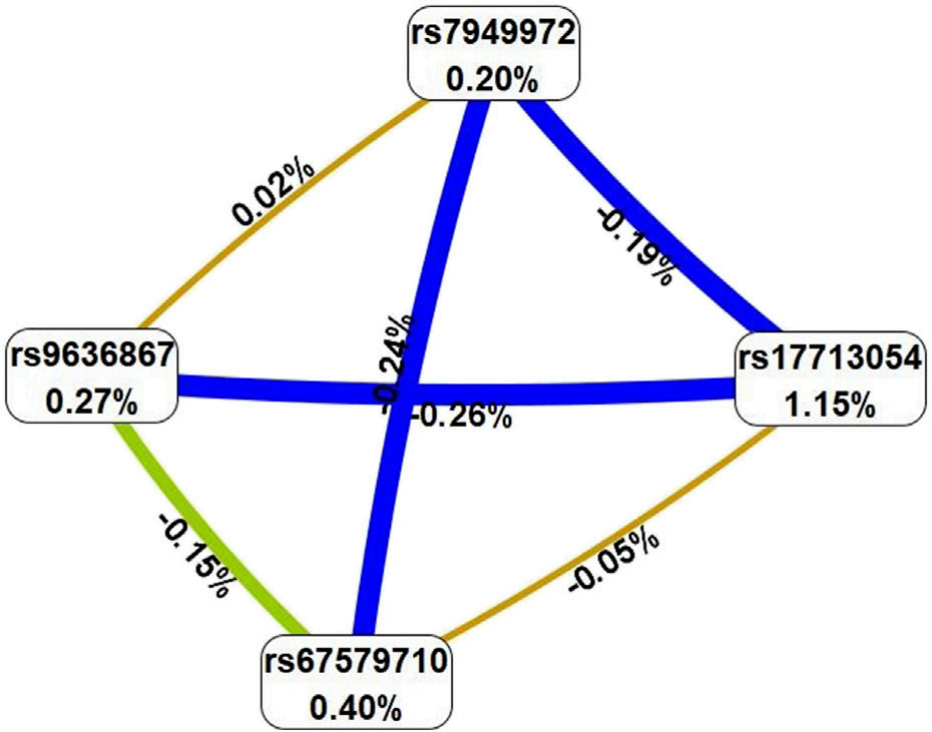
Graph reflecting the structure and strength of the most significant G×G interactions of GWAS-significant loci associated with severe COVID-19. (Note: the color of the lines reflects the nature of the interaction: red and orange lines mean pronounced and moderate synergism, brown means additive effect of genes (independent effects); % reflects the strength and direction of the phenotypic effect of gene interaction (% entropy)).

Using the MB-MDR method, five most significant models of intergenic interactions associated with the severe course of COVID-19 were established: one two-locus model, three three-locus and one four-locus models (*p*_perm_ ≤ 0.001) (Table 2). In total, the best models of G×G interactions included eight polymorphic loci, four of which—rs67579710 *THBS3*, *THBS3*-*AS1*, rs17713054 *SLC6A20-LZTFL1*, rs7949972 *ELF5,* rs9636867 *IFNAR2*—were involved in 2 or more of the most significant G×G interactions. We analyzed the interactions of these genetic variants using the MDR method (Figure 2).

**TABLE 2:** Gene-gene interactions associated with severe COVID-19 (MB-MDR modeling).

Gene-gene interaction models	NH	beta H	WH	NL	beta L	WL	Wmax	*p*_perm_
The best two-locus models of intergenic interactions (for models with Pmin. < 0.001, 1,000 permutations)								
rs67579710 *THBS3*, *THBS3*-*AS1* × rs17713054 *SLC6A20-LZTFL1*	1	0.1538	15.21	1	−0.05222	2.886	15.21	0.002
The best three-locus models of intergenic interactions (for models with Pmin. < 1 × 10^−4^, 1,000 permutations)								
rs67579710 *THBS3*, *THBS3*-*AS1* × rs17713054 *SLC6A20-LZTFL1*× rs143334143 *CCHCR1*	2	0.1673	17.60	1	−0.05878	4.001	17.60	0.005
rs7949972 *ELF5* × rs67579710 *THBS3*, *THBS3*-*AS1* × rs12610495 *DPP9*	3	0.1370	19.41	2	−0.08620	6.019	19.41	0.008
rs9636867 *IFNAR2* × rs67579710 *THBS3*, *THBS3*-*AS1* × rs17713054 *SLC6A20-LZTFL1*	2	0.2242	18.84	1	−0.05784	3.391	18.84	0.018
The best four-locus models of gene-gene interactions (for models with Pmin. < 1 × 10^−5^, 1,000 permutations)								
rs7949972 *ELF5* × rs9636867 *IFNAR2* × rs67579710 *THBS3*, *THBS3*-*AS1* × rs17713054 *SLC6A20-LZTFL1*	4	0.1990	24.34	2	−0.11414	7.205	24.34	0.046

Note: NH, is the number of interacting high-risk genotypes; beta H—regression coefficient for high-risk interactions identified at the second stage of analysis; WH, Wald statistics for high-risk interactions; NL, number of interacting low-risk genotypes; beta L—regression coefficient for low-risk interactions identified at the second stage of analysis; WL, Wald statistics for low-risk interactions; *p*_perm_—permutational significance levels for models (all models are adjusted for gender and age); Loci included in 2 or more best G×G models are indicated in bold.

The MDR method, firstly, showed that the genetic variants included in the best G×G models are characterized by antagonism/additive (independent) effects. Secondly, the mono-effects of SNPs are comparable to the effects of gene-gene interactions in terms of their contribution to the entropy of COVID-19, with the exception of rs17713054, which showed the most prominent mono-effect (1.15%). Thirdly, combinations of genotypes of GWAS-significant SNPs associated with severe COVID-19 are listed in Supplementary Table S4. The combinations with the most pronounced associations with severe COVID-19 are as follows: rs67579710 *THBS3, THBS3-AS1* G/G×rs17713054 *SLC6A20-LZTFL1* A/G (Beta = 0.15378, *p* = 0.0001); rs67579710 *THBS3, THBS3-AS1* G/G×rs17713054 *SLC6A20-LZTFL1* A/G×rs143334143 *CCHCR1* G/G (Beta = 0.16359, *p* = 0.0002 rs7949972 *ELF5* T/C×rs67579710 *THBS3, THBS3-AS1* G/G×rs12610495 *DPP9* G/A) (Beta = 0.11149, *p* = 0.01); rs9636867 *IFNAR2* G/G×rs67579710 *THBS3, THBS3-AS1* G/G×rs17713054 *SLC6A20-LZTFL1* A/G (Beta = 0.215831, *p* = 0.0003); rs7949972 *ELF5* T/C×rs9636867 *IFNAR2* G/G×rs67579710 *THBS3, THBS3-AS1* G/G×rs17713054 *SLC6A20-LZTFL1* A/G (Beta = 0.278009, *p* = 0.002) (Supplementary Table S5).

### 3.3 Environmental-associated correlates of GWAS SNPs

GWAS SNPs were assessed for their potential contribution to COVID-19 severity in combination with environmental risk factors such as smoking, fresh fruit and vegetable consumption, and physical activity level (Supplementary Table S5). SNP rs17713054 *SLC6A20*-*LZTFL1* was associated with an increased risk of severe COVID-19 risk among nonsmokers (risk allele A; OR = 1.65, 95% CI = 1.11–2.46, *p* = 0.02), patients with low fruit and vegetable intake (OR = 1.72, 95% CI = 1.15–2.58, *p* = 0.01, *p*_bonf_ = 0.02), and patients with low levels of physical activity (OR = 1.93, 95% CI = 1.26–2.94, *p* = 0.0035, *p*_bonf_ = 0.007) (Supplementary Table S6).

**FIGURE 3:**
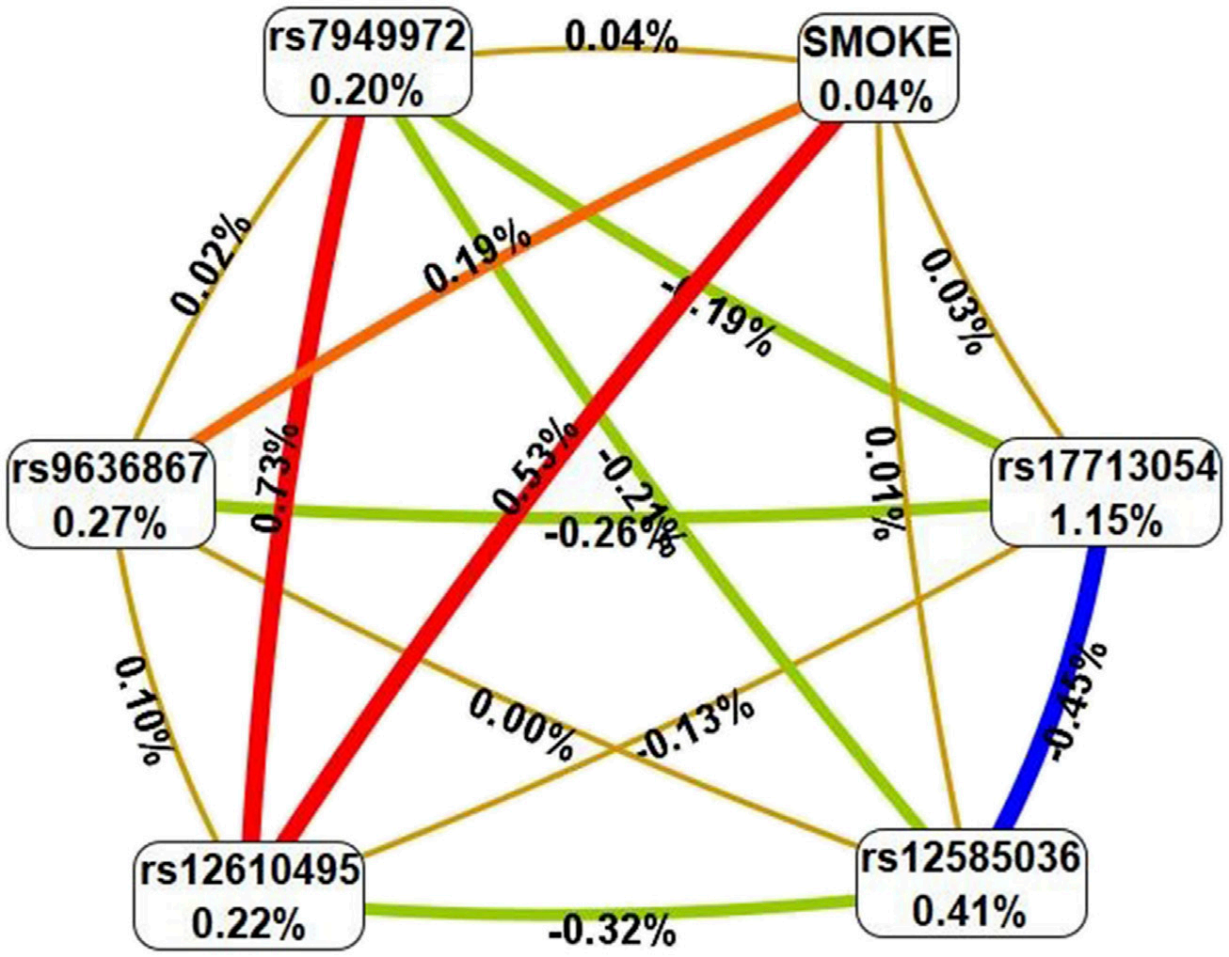
Graph reflecting the structure and power of the most significant G×E interactions of GWAS loci associated with severe COVID-19. (Note: The color of the lines reflects the nature of the interaction: red means strong synergism, brown means additive (independent) effects, and % reflects the strength and direction of the phenotypic effect of gene-environmental interaction (% of entropy)).

Using the MB-MDR approach, the eight most significant models of gene-environment interactions associated with severe COVID-19 were identified: two two-level model, two three-order models, and four four-level models (*p*_perm_ ≤ 0.01) (Table 3). In total, the best G×E models included smoking in interaction with seven loci, five of which—rs7949972 *ELF5*, rs17713054 *SLC6A20*-*LZTFL1*, rs9636867 *IFNAR2*, rs12585036_ATP11A, rs12610495*_DPP9*—were involved in two or more of the most significant G×E interactions. In the next step, we analyzed the interactions between these genetic variants and smoking using the multivariate dimensionality reduction (MDR) method (Figure 3).

**TABLE 3:** Gene-environmental interactions, associated with severe COVID-19 (MB-MDR modeling).

Gene-gene interaction models	NH	beta H	WH	NL	beta L	WL	Wmax	*p*_perm_
The best two-order models of gene-smoking interactions (for G×E models with Pmin. < 0.005, 1,000 permutations)								
SMOKE × rs17713054 *SLC6A20-LZTFL1*	2	0.10911	8.737	1	−0.06466	4.696	8.737	0.02
SMOKE × rs9636867 *IFNAR2*	2	0.11407	7.307	1	−0.08059	3.742	7.307	0.048
The best three-order models of gene-smoking interactions (for G×E models with Pmin. < 0.005, 1,000 permutations)								
SMOKE × rs67579710 *THBS3*, *THBS3*-*AS1* × rs17713054 *SLC6A20-LZTFL1*	2	0.17901	14.892	2	−0.06870	5.292	14.892	0.009
SMOKE × rs9636867 *IFNAR2* × rs12585036 *ATP11A*	2	0.19410	13.116	0	NA	NA	13.116	0.045
The best four-order models of gene-smoking interactions (for G×E models with Pmin. < 1 × 10^−5^, 1,000 permutations)								
SMOKE × rs7949972 *ELF5* × rs9636867 *IFNAR2* × rs17713054 *SLC6A20-LZTFL1*	4	0.3663	25.92	2	−0.12671	8.166	25.92	0.018
SMOKE × rs9636867 *IFNAR2* × rs12585036 *ATP11A* × rs17713054 *SLC6A20-LZTFL1*	6	0.2709	27.16	1	−0.12411	4.163	27.16	0.024
SMOKE × rs9636867 *IFNAR2* × rs12610495 *DPP9* × rs17713054 *SLC6A20-LZTFL1*	5	0.2858	25.01	2	−0.13819	8.247	25.01	0.039
SMOKE × rs7949972 *ELF5* × rs12610495 *DPP9* × rs12585036 *ATP11A*	7	0.2118	27.69	2	−0.14928	7.334	27.69	0.045

Note: NH, is the number of high-risk interactions; beta H—regression coefficient for high-risk interactions identified at the second stage of analysis; WH, Wald statistics for high-risk interactions; NL, number of interacting low-risk interactions; beta L—regression coefficient for low-risk interactions identified at the second stage of analysis; WL, Wald statistics for low-risk interactions; *p*_perm_—permutational significance levels for models (all models are adjusted for gender, age); Loci included in 2 or more best G× E models are indicated in bold.

Firstly, MDR revealed that smoking as an environmental risk factor has the least mono-effect (0.04% contribution to the entropy of severe COVID-19). Secondly, the mono-effects of SNPs/smoking (0.04%–1.15%) are comparable to the effects of gene-environment interactions (0.01%–0.53%). Thirdly, rs17713054 has the maximum mono-effect among the SNPs involved in the most significant gene-environment interactions. (1.15% contribution to entropy). Fourthly, smoking is characterized by multidirectional effects in interaction with SNPs included in the best G×E models: pronounced synergism in interaction with rs12610495, moderate synergism in interaction with rs9636867, additive (independent) effects in interaction with rs17713054, rs12585036, rs7949972. Fifth, the interactions between the genetic variants included in the most significant G×E models are antagonistic/independent (additive effects), with the exception of the interactions between rs7949972 and rs12610495, which exhibit pronounced synergism in interaction with each other. Sixthly, the list of models of gene-environment interactions between GWAS SNPs’ genotypes and smoking is presented in Supplementary Table S6. The following gene-smoking interactions show the strongest correlation with severe COVID-19: non-smokers × rs17713054 *SLC6A20-LZTFL1* G/G (Beta = 0.06466, *p* = 0.031); smokers × rs9636867 *IFNAR2* A/A (Beta = 0.179684, *p* = 0.049); non-smokers ×rs67579710 *THBS3, THBS3-AS1* G/G×rs17713054 *SLC6A20-LZTFL1* A/G (Beta = 0.162513, *p* = 6.77 × 10^−5^); non-smokers ×rs7949972 *ELF5* T/C×rs9636867 *IFNAR2* G/G×rs17713054 *SLC6A20-LZTFL1* A/G (Beta = 0.3137824, *p* = 0.002); smokers ×rs9636867 *IFNAR2* A/A×rs12585036 *ATP11A* C/C×rs17713054 *SLC6A20-LZTFL1* G/G (Beta = 0.648395, *p* = 0.0003); smokers ×rs9636867 *IFNAR2* A/A×rs12610495 *DPP9* G/A×rs17713054 *SLC6A20-LZTFL1* G/G (Beta = 0.485735, *p* = 0.003); non-smokers ×rs7949972 *ELF5* C/C ×rs12610495 *DPP9* A/A×rs12585036 *ATP11A* C/T (Beta = 0.157332, *p* = 0.01) (Supplementary Table S7).

### 3.4 Obesity-depended associations of GWAS SNPs with severe COVID-19

Considering the potential impact of BMI, particularly obesity, on the severity of COVID-19, we carried out an analysis of associations of GWAS SNPs with severe COVID-19 in groups of patients stratified by BMI. Among patients with a BMI less than 30 (non-obese patients), the rs7949972 *ELF5* variant was associated with a reduced risk of severe COVID-19 (protective allele T, OR = 0.67, 95% CI = 0.47–0.95, *p* = 0.02, *p*_bonf_ = 0.04) (Table 4). However, in patients with obesity (BMI ≥30), increased risk of severe COVID-19 was observed for the rs17713054 *SLC6A20*-*LZTFL1* (risk allele A, OR = 2.31, 95% CI = 1.52–3.5, *p* = 0.0002, *p*_bonf_ = 0.0004), rs12610495 *DPP9* (risk allele G, OR = 1.48, 95% CI = 1.09–2.01, *p* = 0.01, *p*_bonf_ = 0.02), and rs17078346 *SLC6A20*-*LZTFL1* (risk allele C, OR = 1.72, 95% CI = 1.15–2.58, *p* = 0.01, *p*_bonf_ = 0.02) (Table 4).

**TABLE 4:** Results of the analysis of associations between GWAS SNPs and severe COVID-19 in obese and non-obese patients.

Genetic variant	Effect allele	Other allele	N	OR [95% CI]^1^	*p* ^2^ (*p*_bonf_)	N	OR [95% CI]^1^	*p* ^2^ (*p*_bonf_)
			BMI <30			BMI ≥30		
rs143334143 *CCHCR1*	A	G	657	0.82 [0.48–1.38]	0.44 (0.88)	658	1.12 [0.70–1.80]	0.63 (1.26)
rs111837807 *CCHCR1*	C	T	656	0.65 [0.36–1.18]	0.14 (0.28)	657	1.09 [0.67–1.78]	0.73 (1.46)
rs17713054 *SLC6A20-LZTFL1*	A	G	657	1.14 [0.69–1.88]	0.61 (1.22)	659	**2.31 [1.52–3.50]**	**0.0002 (0.0004)**
rs17078346 *SLC6A20-LZTFL1*	C	A	657	1.02 [0.64–1.63]	0.93 (1.86)	660	**1.72 [1.15–2.58]**	**0.01 (0.02)**
rs12585036 *ATP11A*	T	C	654	0.80 [0.55–1.16]	0.23 (0.46)	656	0.85 [0.60–1.22]	0.38 (0.76)
rs12610495 *DPP9*	G	A	655	0.85 [0.61–1.19]	0.34 (0.68)	656	**1.48 [1.09–2.01]**	**0.01 (0.02)**
rs7949972 *ELF5*	T	C	647	**0.67 [0.47–0.95]**	**0.02 (0.04)**	650	1.13 [0.82–1.55]	0.46 (0.92)
rs61882275 *ELF5*	A	G	656	0.91 [0.66–1.26]	0.56 (1.12)	657	**1.40 [1.02–1.91]**	**0.036** (0.072)
rs67579710 *THBS3*, *THBS3*-*AS1*	A	G	653	0.79 [0.44–1.42]	0.42 (0.84)	657	0.75 [0.42–1.35]	0.33 (0.66)
rs9636867 *IFNAR2*	G	A	655	0.82 [0.59–1.16]	0.26 (0.52)	657	1.02 [0.74–1.42]	0.91 (1.82)

All calculations were performed relative to the minor alleles (Effect allele); 1 - odds ratio and 95% confidence interval; 2– *p*-value; statistically significant differences are marked in bold.

### 3.5 Relationship between GWAS- significant loci and the clinical characteristics of severe COVID-19 patients

**FIGURE 4:**
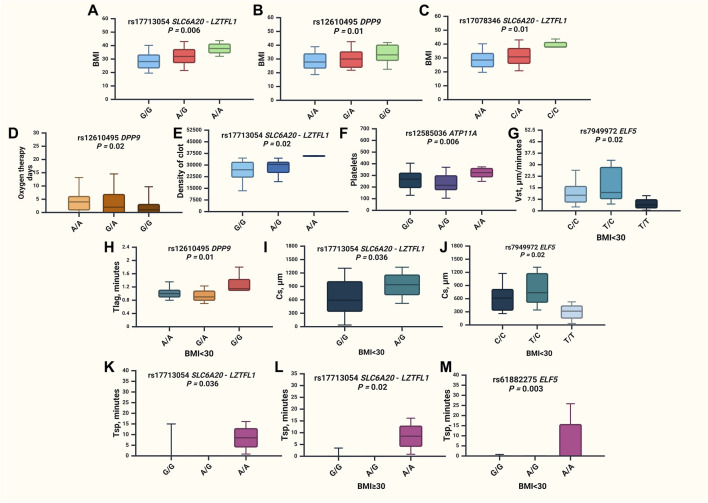
Associations of GWAS loci and clinical characteristics of severe COVID-19 patients. **(A)** BMI values for rs17713054 *SLC6A20-LZTFL1* in the entire group (*p* = 0.006), **(B)** BMI values for rs12610495 *DPP9* in the entire group (*p* = 0.01), **(C)** BMI values for rs17078346 *SLC6A20-LZTFL1* in the entire group (*p* = 0.01), **(D)** oxygen therapy days for rs12610495 *DPP9* in the entire group (*p* = 0.02), **(E)** maximum optical density of the formed clot **(D)** values for rs17713054 *SLC6A20-LZTFL1* in the entire group (*p* = 0.02), **(F)** platelets count for rs12585036 *ATP11A* in the entire group (*p* = 0.006), **(G)** stationary spatial clot growth rates (Vst, μm/minutes) values for rs7949972 *ELF5* in the group of patients with BMI <30 (*p* = 0.02), **(H)** time to the start of clot growth (Tlag, minutes) for rs12610495 *DPP9* in the group of patients with BMI <30 (*p* = 0.01), **(I)** clot size at 30 min post-coagulation activation (CS, μm) values for rs17713054 *SLC6A20-LZTFL1* in the group of patients with BMI <30 (*p* = 0.036), **(J)** clot size at 30 min post-coagulation activation (CS, μm) values for rs7949972 *ELF5* in the group of patients with BMI <30 (*p* = 0.02), **(K)** time of appearance of spontaneous clots (Tsp) values for rs17713054 *SLC6A20-LZTFL1* in the entire group of patients (*p* = 0.036), **(L)**—Tsp values for rs17713054 *SLC6A20-LZTFL1* in the group of patients with BMI<30 (*p* = 0.02), **(M)**—Tsp values for rs61882275 *ELF5* in the group of patients with BMI <30 (*p* = 0.003).

The results of the associations between GWAS SNPs and clinical characteristics of severe COVID-19 patients are presented in Figure 4 and Supplementary Table S8.

Upon the analysis of clinical characteristics among hospitalized COVID-19 patients, it was observed that rs17713054 *SLC6A20-LZTFL1* (*p* = 0.006), rs12610495 *DPP9* (*p* = 0.01), and rs17078346 *SLC6A20-LZTFL1* (*p* = 0.01) were found to be linked with increased BMI (Supplementary Table S7; Figures 4A–C). Additionally, rs12610495 *DPP9* correlated with a reduction in the duration of oxygen therapy (Figure 4D). The maximum optical density of the formed clot (D) was associated with rs17713054 *SLC6A20-LZTFL1* (*p* = 0.02) (Figure 4E). SNP rs12585036 *ATP11A* (*p* = 0.006) increased the count of platelets (Figure 4F). Meanwhile, SNP rs7949972 *ELF5* (*p* = 0.02) reduced in stationary spatial clot growth rates (Vst, μm/minutes) (Figure 4G), rs12610495 *DPP9* (*p* = 0.01) increased the time to the start of clot growth (Tlag, minutes) (Figure 4H), while rs17713054 *SLC6A20-LZTFL1* (*p* = 0.036) and rs7949972 *ELF5* (*p* = 0.02) decreased the clot size at 30 min post-coagulation activation (CS, μm) (Figure 4I, J respectively). Notably, the time of appearance of spontaneous clots (Tsp) was extended in the overall patient group with rs17713054 *SLC6A20-LZTFL1* (*p* = 0.0036) (Figure 4K). Given the strong correlation between rs17713054 *SLC6A20-LZTFL1*, rs12610495 *DPP9*, and rs17078346 *SLC6A20-LZTFL1* with BMI, we conducted a comparison of clinical characteristics between two patient groups based on BMI status. In patients with a BMI ≥30, SNP rs17713054 *SLC6A20-LZTFL1* (Figure 4L) was associated with an elevation in Tsp (*p* = 0.02), while among patients without obesity (BMI <30) rs61882275 *ELF5* (*p* = 0.003) was found to increase Tsp (Figure 4M).

### 3.6 Functional annotation of severe COVID-19-related SNPs

#### 3.6.1 QTL-effects

The results of the cis-eQTL analysis (Table 5) shed light on the impact of specific genetic variants on gene expression. According to the eQTLGen Browser, rs17713054 *SLC6A20*-*LZTFL1* and rs17078346 *SLC6A20*-*LZTFL1* were associated with a decrease in the expression of *FLT1P1*, *CCR3*, *CCR1*, *SACM1L*, *CCR5*, *CCR2*, *RP11*-*24F11.2*, and *CXCR6*, while these two SNPs were linked to an increase in the expression of CCR9 in blood. Moreover, data from the GTEx Portal indicated that rs17713054 *SLC6A20*-*LZTFL1* was associated with reduced expression levels of *CXCR6* in tibial artery and adipose tissues (subcutaneous), alongside an elevation in the expression of *LZTFL1* in adipose tissue (subcutaneous).

**TABLE 5:** Association of SNPs with cis-eQTL-Mediated Expression Profiles of GWAS Genes.

		eQTLGen Browser data			GTEx Portal data			
SNP	Effect allele	Gene expressed	Z-score	*p*-value	Gene expressed	*p*-value	Effect (NES)	Tissue
rs17713054 *SLC6A20-LZTFL1*	A	*CXCR6*	↓(−13.9294)	4.20 × 10^−44^	*CXCR6*	1.7 × 10^−7^	↓(-0.42)	Artery - Tibial
		*FLT1P1*	↓(−15.1094)	1.40 × 10^−51^				
		*CCR3*	↓(−14.3393)	1.24 × 10^−46^				
		*CCR9*	↑(5.1055)	3.30 × 10^−7^	*CXCR6*	6.7 × 10^−5^	↓(-0.30)	Adipose - Subcutaneous
		*CCR1*	↓(−7.4173)	1.19 × 10^−13^				
		*SACM1L*	↓(−5.7667)	8.08 × 10^−9^	*LZTFL1*	1.0 × 10^−4^	↑(0.21)	Adipose - Subcutaneous
		*CCR5*	↓(−5.206)	1.93 × 10^−7^				
		*CCR2*	↓(−4.9694)	6.71 × 10^−7^	*CCR9*	1.6 × 10^−4^	↑(0.33)	Whole Blood
		*RP11-24F11.2*	↓(−4.6342)	3.58 × 10^−6^				
rs17078346 *SLC6A20-LZTFL1*	С	*CCR3*	↓(−13.0025)	1.18 × 10^−38^	-			
		*FLT1P1*	↓(−12.847)	8.94 × 10^−38^				
		*CXCR6*	↓(−11.6247)	3.08 × 10^−31^				
		*CCR1*	↓(−6.5185)	7.10 × 10^−11^				
		*CCR5*	↓(−5.5666)	2.59 × 10^−8^				
		*SACM1L*	↓(−5.4113)	6.25 × 10^−8^				
		*CCR2*	↓(−5.3267)	9.99 × 10^−8^				
		*CCR9*	↑(5.0282)	4.95 × 10^−7^				
		*RP11-24F11.2*	↓(−4.5892)	4.44 × 10^−6^				
rs12585036 *ATP11A*	T	*ATP11A*	↓(−7.9284)	2.22 × 10^−15^	*ATP11A*	7.3 × 10^−8^	↓(−0.18)	Artery - Aorta
					*RP11-88E10.5*	2.2 × 10^−6^	↓(−0.34)	Artery - Coronary
rs12610495 *DPP9*	G	*DPP9*	↓(−14.4364)	3.05 × 10^−47^	*DPP9*	4.50 × 10^−9^	↓(−0.18)	Lung
		*TNFAIP8L1*	↑(7.6938)	1.43 × 10^−14^	*DPP9*	8.90 × 10^−8^	↓(−0.15)	Artery - Tibial
					*DPP9*	4.1 × 10^−6^	↓(−0.17)	Artery - Aorta
rs7949972 *ELF5*	T	*CAT*	↓(−56.0274)	3.27 × 10^−310^	*ELF5*	2.50 × 10^−15^	↓(−0.23)	Lung
					*CAT*	3.20 × 10^−14^	↓(−0.25)	Whole Blood
		*ABTB2*	↓(-15.164)	6.12 × 10^−52^	*CAT*	4.3 × 10^−6^	↓(−0.15)	Artery - Tibial
					*ABTB2*	0.00007	↓(−0.17)	Whole Blood

Additionally, rs12585036 *ATP11A* was correlated with decreased expression levels of *ATP11A* in the blood and aorta, as well as *RP11-88E10.5* in coronary arteries. rs12610495 DPP9 showed associations with reduced expression of *DPP9* in blood, lung, and arteries (tibial artery and aorta), while it was linked to an increase in the expression levels of *TNFAIP8L1* in blood. Notably, rs7949972 *ELF5* demonstrated a decrease in expression levels of *CAT* in whole blood and artery (tibial), while *ABTB2* expression was reduced solely in whole blood by the influence of this SNP. Furthermore, *ELF5* expression was found to be decreased in the lungs, indicating the effects of rs7949972.

#### 3.6.2 Histone modifications

Using the bioinformatics tool HaploReg (v4.2), we analyzed histone modifications associated with SNPs identified in our study as linked to an increased risk of severe COVID-19 (Table 6).

**TABLE 6:** The impact of GWAS SNPs on histone tags in various tissues.

SNP (Ref/Alt allele)	Tissues Marks	Lung	Vessels—aorta	Blood	Adipose tissue
rs17713054 (G/A) *SLC6A20*-*LZTFL1*	H3K4me1	Enh	Enh	-	Enh
	H3K4me3	-	-	-	-
	H3K27ac	Enh	Enh	-	-
rs12610495 (A/G) *DPP9*	H3K4me1	Enh	-	Enh	-
	H3K4me3	Pro	-	-	-
	H3K27ac	Enh	-	-	-
rs7949972 (C/T) *ELF5*	H3K4me1	Enh	-	-	-

H3K4me1—mono-methylation at the fourth lysine residue of the histone H3 protein; H3K4me3—tri-methylation at the fourth lysine residue of the histone H3 protein; H3K9ac—the acetylation at the ninth lysine residues of the histone H3 protein; H3K27ac—acetylation of the lysine residues at N-terminal position 27 of the histone H3 protein; effect alleles are marked in bold. Enh—histone modification in the enhancer region; Pro—histone modification at the promoter region.

SNP rs17713054 *SLC6A20*-*LZTFL1* is situated in a DNA-binding region associated with histone H3 monomethylation at the fourth lysine residue (H3K4me1) in lung, aorta, and adipose tissue. Moreover, this SNP has further influence on H3K27ac, which marks enhancers, particularly in lung tissues and the aorta.

Similarly, rs12610495 *DPP9* is located in a DNA-binding region associated with H3K4me1 in both the lungs and blood. In lung tissue, it also binds to H3K4me3. Additionally, the impact of these histone modifications is further enhanced by the presence of H3K27ac.

Finally, rs7949972 *ELF5* falls within a region of DNA binding to H3K4me1 exclusively in lung tissue.

#### 3.6.3 Analysis of transcription factors

The risk allele A of rs17713054 *SLC6A20-LZTFL1* is associated with the generation of DNA binding sites for 48 transcription factors (TFs) (Supplementary Table S9). These TFs are involved in four overrepresented biological processes: integrated stress response signaling (GO:0140467; FDR = 1.48 × 10^−12^), positive regulation by host of viral transcription (GO:0043923; FDR = 4.68 × 10^−2^), fat cell differentiation (GO:0045444; FDR = 3.45 × 10^−4^), transforming growth factor beta receptor signaling pathway (GO:0007179; FDR = 4.94 × 10^−2^). The protective allele G of rs17713054 *SLC6A20-LZTFL1* creates binding sites for 24 TFs, jointly involved in response to hypoxia (GO:0001666; FDR = 2.8 × 10^−2^).

The protective allele T rs12585036 *ATP11A* generates DNA binding sites for 104 TFs (Supplementary Table S10) involved in response to (GO:1990785; FDR = 8.08 × 10^−3^), response to testosterone (GO:0033574; FDR = 4.09 × 10^−11^), androgen receptor signaling pathway (GO:0030521; FDR = 8.49 × 10^−3^), canonical Wnt signaling pathway (GO:0060070; FDR = 3.54 × 10^−3^).

As for the risk allele C rs17078346 *SLC6A20-LZTFL1,* it creates DNA binding regions for 31 TFs (Supplementary Table S11), that are involved in three overrepresented biological processes: epithelial tube branching involved in lung morphogenesis (GO:0060441; FDR = 7.59 × 10^−4^), Notch signaling pathway (GO:0007219; FDR = 1.29 × 10^−3^).

Protective allele A rs12610495 *DPP9* is associated with the generation of DNA binding sites for 39 TFs (Supplementary Table S12). These TFs jointly participate in positive regulation of regulation of cytokine production (GO:0001817; FDR = 0.0475).

Finally, risk allele C rs7949972 *ELF5* creates DNA binding sites for 32 TFs (Supplementary Table S13), that are jointly involved in the following overrepresented biological processes: positive regulation of CD8-positive, alpha-beta T cell differentiation (GO:0043378; FDR = 0.00247), negative regulation of CD4-positive, alpha-beta T cell differentiation (GO:0043371; FDR = 0.0301), defense response to virus (GO:0051607; FDR = 0.00177), positive regulation of interferon-alpha production (GO:0032727; FDR = 0.0413), positive regulation of interferon-beta production (GO:0032728; 0.00251).

#### 3.6.4 Bioinformatic analysis of the associations of GWAS SNPs with COVID-19-related phenotypes

According to the bioinformatic resource Lung Disease Knowledge Portal, the GWAS SNPs rs17713054, rs12585036, rs17078436, rs12610495 are linked to the higher risk of hospitalization of COVID-19 patients and to the severe respiratory confirmed COVID-19. Additionally, rs12585036 is associated with a reduction in lung capacity parameters such as forced vital capacity (FVC), forced expired volume in 1 s (FEV1), FEV1 to FVC ratio, peak expiratory flow. Conversely, rs7949972 is associated with a lower risk of hospitalization in COVID-19 patients while increasing the lung capacity parameters (Table 7).

**TABLE 7:** Results of aggregated bioinformatic analyzes of associations between GWAS SNPs and the risk of severe COVID-19 course.

No	SNP	Phenotype	*p*-value	Beta (OR)	Sample size
1	rs17713054 *SLC6A20-LZTFL1* (G/A)	Very severe respiratory confirmed COVID-19 vs. population	1.15 × 10^−80^	_OR_▲1.8111	7,252
2		Hospitalized COVID-19 vs. population	1.09 × 10^−51^	_OR_▲1.8134	908,494
3		Hospitalized vs. non-hospitalized COVID-19	2.04 × 10^−28^	_OR_▲1.3555	10,216
4		COVID-19 vs. population	4.80 × 10^−26^	_OR_▲1.3121	1,299,010
5		Very severe respiratory confirmed vs. non-hospitalized COVID-19	1.43 × 10^−5^	_OR_▲2.8766	957
6		COVID-19 vs. no COVID-19	3.12 × 10^−5^	_OR_▲1.1324	127,879
7	rs12585036 *ATP11A* (C/T)	Idiopathic pulmonary fibrosis	2.36 × 10^−16^	_OR_▼0.9994	57,913
8		FEV1 to FVC ratio	1.97 × 10^−6^	_Beta_▼-0.0141	793,368
9		Very severe respiratory confirmed COVID-19 vs. population	8.12 × 10^−6^	_OR_▲1.1025	7,376
10		Forced expired volume in 1 s (FEV1)	8.26 × 10^−6^	_Beta_▼-0.0128	793,442
11		Peak expiratory flow	5.68 × 10^−4^	_Beta_▼-0.0105	690,530
12		Hospitalized vs. non-hospitalized COVID-19	0.002	_OR_▲1.0604	10,013
13		Hospitalized COVID-19 vs. population	0.004	_OR_▲1.0604	908,494
14		Forced vital capacity (FVC)	0.025	_Beta_▼-0.0063	792,938
15		Airway wall area in COPD	0.029	_Beta_▼-0.0334	12,031
16	rs17078346 *SLC6A20-LZTFL1* (A/C)	Very severe respiratory confirmed COVID-19 vs. population	2.96 × 10^−39^	_OR_▲1.5011	5,855
17		Hospitalized COVID-19 vs. population	1.08 × 10^−18^	_OR_▲1.4711	898,438
18		Hospitalized vs. non-hospitalized COVID-19	1.01 × 10^−16^	_OR_▲1.2208	10,256
19		COVID-19 vs. population	3.57 × 10^−9^	_OR_▲1.1637	1,288,650
20		COVID-19 vs. no COVID-19	8.72 × 10^−5^	_OR_▲1.1040	127,879
21		Very severe respiratory confirmed vs. non-hospitalized COVID-19	2.42 × 10^−4^	_OR_▲2.2105	957
22	rs12610495 *DPP9* (A/G)	Very severe respiratory confirmed COVID-19 vs. population	1.64 × 10^−15^	_OR_▲1.2015	5,642
23		Idiopathic pulmonary fibrosis	4.11 × 10^−15^	_OR_▲1.0003	58,925
24		Hospitalized COVID-19 vs. population	4.84 × 10^−8^	_OR_▲1.1914	895,822
25		Hospitalized vs. non-hospitalized COVID-19	1.73 × 10^−5^	_OR_▲1.0769	9,939
26		COVID-19 vs. population	1.64 × 10^−4^	_OR_▲1.0704	1,274,140
27		COVID-19 vs. no COVID-19	0.0025	_OR_▲1.0603	101,592
28	rs7949972 *ELF5* (C/T)	Very severe respiratory confirmed COVID-19 vs. population	6.47 × 10^−7^	_OR_▼0.9079	7,225
29		FEV1 to FVC ratio	3.9 × 10^−6^	_Beta_▼-0.0112	808,254
30		Hospitalized vs. non-hospitalized COVID-19	7.01 × 10^−5^	_OR_▼0.9392	10,256
31		Hospitalized COVID-19 vs. population	0.0021	_OR_▼0.9254	905,878
32		Forced vital capacity (FVC)	0.0034	_Beta_▲0.0071	807,822
33		COVID-19 vs. population	0.022	_OR_▼0.9642	1,289,590
34		Emphysema in COPD (percentage low attenuation area −950 HU)	0.024	_Beta_▲0.0375	12,031
35		Emphysema in COPD (15th percentile HU)	0.048	_Beta_▼-0.0245	12,031

data obtained using the bioinformatic resource Lung Disease Knowledge Portal https://lung.hugeamp.org/

Effect alleles are marked in bold.

## 4 Discussion

In the present study, we replicated associations of the rs17713054 *SLC6A20*-*LZTFL1*, rs17078346 *SLC6A20*-*LZTFL1*, rs12610495 *DPP9* and rs7949972 *ELF5* with severe COVID-19 within the Caucasian population of Central Russia. For the first time in the world, we assessed the impact of COVID-19 GWAS loci on a wide range of clinical manifestations of the disease, primarily on thrombodynamic parameters, identified the most significant intergenic interactions, and also assessed how environmental risk factors and obesity modify associations of GWAS loci with the risk of severe COVID-19; conducted a comprehensive functional annotation of severe COVID-19-associated SNPs to analyse their involvement in the molecular mechanisms of the disease.

**FIGURE 5:**
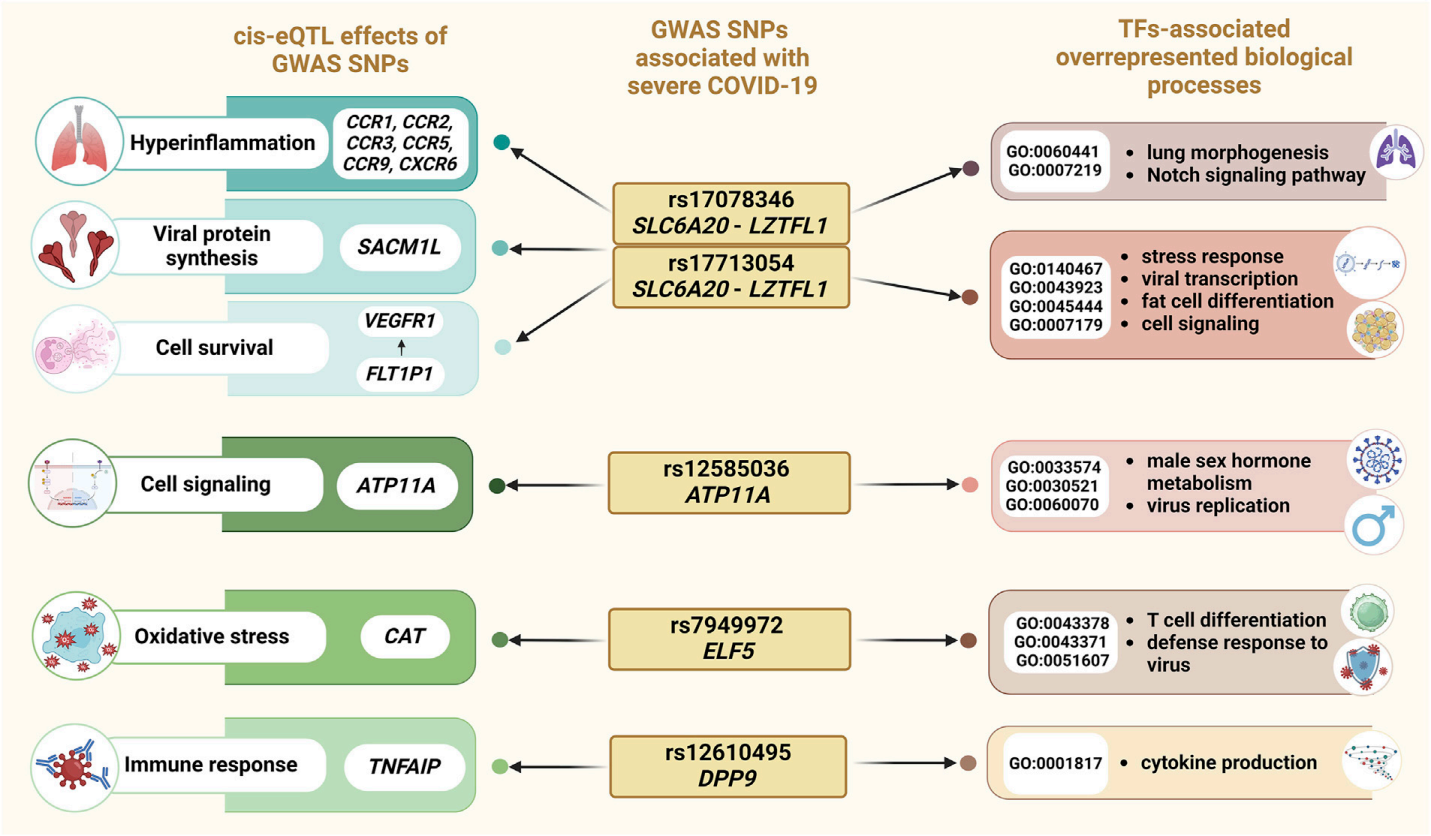
Overview of the results of an integrated bioinformatics investigation of severe COVID-19-associated SNPs.

Figure 5 summarizes the principal molecular mechanisms underlying the involvement of GWAS SNPs to severe COVID-19.

First of all, we identified that both studied polymorphic variants located in the *SLC6A20-LZTFL1* region are associated with COVID-19: rs17713054 *SLC6A20-LZTFL1* (risk allele A) increases the risk of severe COVID-19 regardless of sex and age; however, this risk can be modified by smoking status, intake of fresh fruit and vegetables, and higher levels of physical activity. Moreover, rs17713054 (risk allele A) was found to be associated with an increase in body mass index and worsening thrombodynamic parameters, including an increase in the maximum optical density of the formed clot (D), delayed appearance of spontaneous clots (Tsp), and larger clot size 30 min after coagulation activation (CS). It is noteworthy that rs17713054 showed an association with severe COVID-19 in a large number of replication studies conducted around the world (Roberts et al., 2020; Downes et al., 2021; Roozbehani et al., 2023; Udomsinprasert et al., 2023). However, the possible influence of rs17713054 on both the development of COVID-19 and the development of coronary artery disease is a topic of active discussion in the literature (Wang et al., 2023). According to our mediation analysis, the contribution of rs17713054 to SARS-CoV-2 susceptibility may be mediated through comorbid disease in severe COVID-19 patients, to a lesser extent by chronic obstructive pulmonary disease, and to a greater extent by coronary artery disease.

SNP rs17078346 *SLC6A20-LZTFL1* (risk allele C) also was associated with the increased the risk of severe COVID-19 in our study, exclusively in obese patients. Possible molecular mechanisms of the involvement of these genetic variants in the risk of developing severe COVID-19 may be associated with their regulation of the *LZTFL1* gene (Leucine Zipper Transcription Factor Like 1), which regulates protein trafficking to the ciliary membrane, the violation of which may play an important role in weakened airway viral clearance in a patient with COVID-19 (Robinot et al., 2021). Moreover, *LZTFL1* regulates the transition of epithelial cells to mesenchymal cells (https://www.genecards.org/cgi-bin/carddisp.pl?gene=LZTFL1), thereby participating in the regulation of the viral response pathway associated with epithelial-mesenchymal transition (Downes et al., 2021), an important regulator of the innate immune response.

Our bioinformatic analysis revealed that allele A rs17713054 *SLC6A20-LZTFL1* and allele C rs17078346 *SLC6A20-LZTFL1* influence the expression of other genes through cis-eQTL-effects: these SNPs are associated with a decrease in the expression of *FLT1P1* in blood, potentially resulting in dysregulation of vascular endothelial growth factor receptor 1 (VEGFR1) expression (Ye et al., 2015). Numerous studies have demonstrated a correlation between elevated VEGFR1 levels and COVID-19 severity, as well as the ICU admission of COVID-19 patients (Krock et al., 2011; Ackermann et al., 2020; Nagashima et al., 2020; Pine et al., 2020; Miggiolaro et al., 2023; Pius-Sadowska et al., 2023). In addition, we noted eQTL effects of rs17713054 and rs17078346 on the expression levels of chemokine receptors (*CCR1, CCR2, CCR3, CCR5, CCR9,* and *CXCR6*). Previous studies have implicated these chemokine receptors in virus infections and COVID-19 pathogenesis, suggesting their role in lung infiltration by monocytes and macrophages during viral infection, contributing to the hyperinflammation observed in severe COVID-19 cases (Coperchini et al., 2020; Khalil et al., 2021; Mahmoodi et al., 2024). Among other genes with altered expression levels caused by rs17713054 and rs17078346 is *SACM1L,* which was previously identified as a putative causal gene for COVID-19 severity (Wu et al., 2021). SACM1L mediates lipid transfer between closely opposed ER and endosomal membranes with several other lipid transfer proteins (Reinisch and Prinz, 2021). It was found that SACM1L concentrated at the viral factories in infected cells, contrasting its typical distribution in uninfected cells, where it is primarily found in the ER and Golgi apparatus (García-Dorival et al., 2023) (Figure 5).

TFs binding to the risk allele A rs17713054 are associated with positive regulation by host of viral transcription (GO:0043923), integrated stress response signaling (GO:0140467), the transforming growth factor beta receptor signaling pathway (GO:0007179), and fat cell differentiation (GO:0045444), while also resulting in a loss of function in response to hypoxia (GO:0001666). These findings provide insights into the association of rs17713054 with severe COVID-19 and obesity, a known risk factor for severe COVID-19 progression. Risk allele C rs17078346 *SLC6A20-LZTFL1* affects DNA binding to TFs jointly involved in epithelial tube branching involved in lung morphogenesis (GO:0060441), and the Notch signaling pathway (GO:0007219) (Figure 5). These findings suggest its potential role in COVID-19 severity by regulating immune response, and apoptosis.

The correlation between rs17713054 *SLC6A20-LZTFL1* and obesity is supported by previous research indicating that *LZTFL1* may regulate leptin signaling, and participate in the LepRb signaling pathway in the hypothalamus, which controls energy homeostasis (Wei et al., 2018). Elevated levels of circulating leptin are generally attributed to the development of leptin resistance (Zieba et al., 2020), a hallmark of obesity, which is already recognized as a risk factor for severe COVID-19 (Rebello et al., 2020; Maurya et al., 2021). Notably, Lztfl1 knockout mice exhibit hyperphagia, leptin resistance, and obesity (Tomlinson, 2024). Moreover, polyphenolic compounds found in fruits and vegetables, along with regular exercise, have been shown to enhance sensitivity to leptin (Aragonès et al., 2016; Fedewa et al., 2018). Based on these findings, we hypothesize that individuals carrying the allele A of rs17713054 *SLC6A20-LZTFL1*, who consume higher levels of fruit and vegetables and engage in more physical activity, may experience reduced inflammation by lowering serum leptin levels, potentially leading to a less severe course of COVID-19. Additionally, the manifestation of the risk effects of rs17713054 *SLC6A20-LZTFL1* in patients with low levels of physical activity may be explained by the significant suppression of *Slc6A20* expression observed in mouse models following exercise (Walz et al., 2021). Considering that *SLC6A20* expression is positively associated with infiltrating neutrophils and immune-related signatures (Acar, 2023), the downregulation of this gene through exercise may further contribute to the mitigation of COVID-19 severity. The presence of the rs17713054 *SLC6A20-LZTFL1* association in patients with low consumption of fresh vegetables and fruits—one of the main environmental risk factors for oxidative stress—may be associated with the effect of reactive oxygen species on the expression level of the *SLC6A20* and *LZTFL1* genes. In particular, it was found that hydrogen peroxide, along with plant extracts, may affect the expression of *SLC6A20* mRNA and *LZTFL1* mRNA (Briedé et al., 2010; Tomé-Carneiro et al., 2013).

The association of rs17713054 *SLC6A20-LZTFL*1 with severe COVID-19 in non-smoking individuals can be explained; on the one hand, smoking itself is a known risk factor for severe COVID-19 due to its upregulation of *ACE-2* expression in the lungs, the host receptor for SARS-CoV-2, making smokers more susceptible to the disease (Reddy et al., 2021). This increased susceptibility to COVID-19 in smokers may exceed the effect of rs17713054, leading to the observed association specifically in non-smokers. On the other hand, previous research has shown that smoking affects the expression of genes located near rs17713054, the level of *SLC6A20* mRNA, and the decreased expression of *LZTFL1* (Xiong et al., 2021). Another study showed that benzopyrene, one of the main components of cigarette smoke, increases methylation of the *LZTFL1* gene promoter and exon *SLC6A20* (Jiang et al., 2017) and also reduces the expression of *SLC6A20* mRNA (Qiu et al., 2011; Kreuzer et al., 2020). Considering that increased methylation is a significant regulatory mechanism for decreased gene expression, this finding can be interpreted as further evidence that smoking influences the decreased expression of *LZTFL1* and *SLC6A20*.

Furthermore, our study showed that rs12610495 *DPP9* (risk allele G) is associated with a higher risk of severe COVID-19 in patients with obesity and also affects BMI in patients with severe COVID-19. Additionally, a significant association was found between rs12610495 and thrombodynamic parameters, in particular with prolongation of the time to the start of clot growth (Tlag). Several studies have already pointed to rs12610495 *DPP9* as a risk polymorphic variant for severe COVID-19 (Degenhardt et al., 2022; Horowitz et al., 2022; Thibord et al., 2022; Pairo-Castineira et al., 2023). *DPP9* plays a diverse role in immune regulation: it participates in the activation of inflammasomes (Okondo et al., 2018), its inhibition induces pro-caspase-1-dependent monocyte and macrophage pyroptosis (Okondo et al., 2017). Knockdown of *Dpp9* significantly impairs preadipocytes differentiation (Han et al., 2015), supporting our findings that rs12610495 *DPP9* associates with BMI. This SNP has a high regulatory potential in lung tissue, being marked by the enhancer tags H3K4me1 and H3K27ac as well as by the promoter tag H3K4me3. The risk allele G rs12610495 *DPP9* disrupts the regulation of cytokine production (GO:0001817), potentially leading to dysregulated production of proinflammatory cytokines. This dysregulation may cause excessive immune cell infiltration in pulmonary tissues, leading to tissue damage (Nagashima et al., 2020). In blood, rs12610495 *DPP9* alters the expression through cis-eQTL effects of *TNFAIP8L1*, a member of the TNFAIP family, which plays a modulating role in immune response (Li et al., 2018; Hua et al., 2021). Additionally, Pahl et al. reported that *TNFAIP8L1* levels were significantly downregulated in monocytes from COVID-19 patients compared to healthy controls (Pahl et al., 2022) (Figure 5).

We determined that rs7949972 *ELF5* (effect allele T) had a protective effect only in COVID-19 patients with a BMI <30. In this subgroup, we observed that the protective allele T reduces the clot size at 30 min after coagulation activation (CS) and stationary spatial clot growth rates (Vst). ELF5, a member of the erythroblast transformation-specific (Ets) transcription factor family, has been extensively studied in breast cancer contexts (Chakrabarti et al., 2012; Kalyuga et al., 2012). However, recent research has highlighted its role in COVID-19, revealing that key host factors for SARS-CoV-2 (*Ace2* and *Tmprss4*) are upregulated in *Elf5*-overexpressing AT2 cells (Pietzner et al., 2022). *ELF5,* through cis-eQTL effects, also regulates the expression of *CAT*, an antioxidant enzyme, in whole blood and in the tibial artery. Levels of catalase, along with other markers of oxidative stress, were found to be elevated in COVID-19 patients (Martín-Fernández et al., 2021). Oxidative stress may not only pose a risk for severe COVID-19 but also contribute to the development of atherosclerosis (Sorokin et al., 2015; Sorokin et al., 2016)and atherosclerosis-associated cardiovascular diseases (Vialykh et al., 2012; Bushueva OY. et al., 2015; Bushueva et al., 2021), exacerbating patient prognosis (Hessami et al., 2021). Upon analyzing the impact of the risk allele C rs7949972 *ELF5* on TFs binding sites, we hypothesize that this allele may result in a more severe COVID-19 course. This could be due to its positive regulation of CD8-positive, alpha-beta T cell differentiation (GO:0043378), and negative regulation of CD4-positive, alpha-beta T cell differentiation (GO:0043371), as well as its involvement in the defense response to viruses (GO:0051607) (Figure 5). These processes may contribute to excessive inflammation and worsen the course of COVID-19. Additionally, data from the Lung Knowledge Portal indicates that protective allele T rs7949972 correlates with an increase in parameters such as forced vital capacity (FVC), forced expired volume in 1 s (FEV1), FEV1 to FVC ratio, and peak expiratory flow.

Finally, allele T rs12585036 *ATP11A* exhibited a protective effect against severe COVID-19, but exclusively in men. The ATPase phospholipid transporting 11A (*ATP11A*) gene encodes a membrane ATPase responsible for translocating phosphatidylserine (PtdSer) (Segawa et al., 2021). Phagocytosis associated with PtdSer translocation could serve as an early event linked to viral infections (Takizawa et al., 1993; Banki et al., 1998). Moreover, PtdSer has been implicated as a potential mechanism or participant in inflammation and coagulation abnormalities in COVID-19 patients (Argañaraz et al., 2020; Wang et al., 2022). We hypothesize that the protective effect of the T allele of rs12585036 *ATP11A* regarding the risk of severe COVID-19 specifically in men is due to the fact that female sex hormones, in particular estradiol, lead to increased expression of *ATP11A* (Logan et al., 2010; Vydra et al., 2019). Considering the fact that the protective T allele is associated with a decrease in *ATP11A* expression through cis-eQTL effects, it can be assumed that the influence of female sex hormones can neutralize this effect by increasing the level of *ATP11A*. Moreover, bioinformatics analysis revealed that the protective T allele rs12585036 creates DNA binding sites for TFs involved in overrepresented biological processes related to male sex hormone metabolism (response to testosterone (GO:0033574) and androgen receptor signaling pathway (GO:0030521)) and regulation of canonical Wnt signaling pathway (GO:0060070; FDR = 3.54 × 10^−3^), which has been shown to inhibit the replication of SARS-CoV-2 *in vitro*, and reduce viral load, inflammation and clinical symptoms in a mouse model of COVID-19 (Xu et al., 2024). This finding suggests a potential explanation for why SNP rs12585036 *ATP11A* protects against COVID-19 in men.

## 5 Study limitations

Firstly, our study was limited in its scope, as we were unable to investigate other genes implicated in the progression of severe COVID-19. Secondly, we lacked data on the vaccination status of the control group, as well as laboratory parameters, including venous blood for thrombodynamics testing, which could only be obtained during hospitalization. This limitation prevented us from conducting a formal comparative analysis of laboratory parameters, incl. thrombodynamic parameters between control group patients and patients with severe COVID-19. Additionally, the effectiveness of different types of vaccines remains controversial, adding further complexity to the analysis of data and the role of vaccination in protecting against severe COVID-19. Thirdly, essential environmental factors such as vegetable intake and physical activity levels were not available for the control group, preventing their inclusion in the MB-MDR analysis of gene-environmental interactions.

## Data Availability

The datasets presented in this study can be found in online repositories. The names of the repository/repositories and accession number(s) can be found in the article/Supplementary Material.
