# Supplementary material for: GWAS-significant loci and severe COVID-19: analysis of associations, link with thromboinflammation syndrome, gene-gene, and gene-environmental interactions
Source: Front Genet. 2024 Aug 8;15:1434681. doi: 10.3389/fgene.2024.1434681 (PMC11338913; doi:10.3389/fgene.2024.1434681)
Supplement: Supplementary file 1 [file Table1.DOCX]

Supplementary Material

**Supplementary Table 1.** Baseline and clinical characteristics of the studied groups.

| Baseline and clinical characteristics | | | COVID-19 patients  (n=199) | Controls  (n=599) | *p*-value |
| --- | --- | --- | --- | --- | --- |
| Age, Ме [Q1; Q3] | | | 68 [57; 78] | 57 [52; 64] | **<0.001** |
| Gender | Males, N (%) | | 92 (46.2%) | 220 (36.7%) | **<0.001** |
|  | Females, N (%) | | 107 (53.8%) | 379 (63.3%) |  |
| Smoking | Yes, N (%) | | 56 (28%) | 175 (29.2%) | **>0.05** |
|  | No, N (%) | | 137 (69%) | 411 (68.6%) |  |
|  | ND, N (%) | | 6 (3%) | 13 (2.2%) |  |
| Low physical activity | Yes, N (%) | | 106 (53.3%) | ND | - |
|  | No, N (%) | | 87 (43.7%) |  |  |
|  | ND, N (%) | | 6 (3%) |  |  |
| Low fruit/vegetable consumption | Yes, N (%) | | 126 (63.3%) | ND | - |
|  | No, N (%) | | 67 (33.7%) |  |  |
|  | ND, N (%) | | 6 (3%) |  |  |
| Vaccination | Yes, N (%) | | 21 (10.6%) | 73 (15.8%) | **>0.05** |
|  | No, N (%) | | 20 (10.1%) | 65 (14.1%) |  |
|  | ND, N (%) | | 146 (73.4%) | 461 (70.1%) |  |
| Death | Yes, N (%) | | 67 (33.7%) | 0 (0%) | **<0.001** |
|  | No, N (%) | | 132 (66.3%) | 599 (100%) |  |
| Essential hypertension (EH) | Yes, N (%) | | 129 (64.8%) | 111 (18.5%) | **<0.001** |
|  | No, N (%) | | 70 (39.7%) | 488 (81.5%) |  |
| Coronary artery disease (CAD) | Yes, N (%) | | 84 (42.2%) | 0 (0%) | **<0.001** |
|  | No, N (%) | | 115 (57.8%) | 599 (100%) |  |
| Cerebrovascular accident (CVA) in anamnesis | Yes, N (%) | | 32 (16.1%) | 0 (0%) | **<0.001** |
|  | No, N (%) | | 167 (83.9%) | 599 (100%) |  |
| Chronic obstructive pulmonary disease (COPD) | Yes, N (%) | | 46 (23.1%) | 0 (0%) | **<0.001** |
|  | No, N (%) | | 153 (76.9%) | 599 (100%) |  |
| Diabetes mellitus type 2 (T2D) | Yes, N (%) | | 39 (19.6%) | 0 (0%) | **<0.001** |
|  | No, N (%) | | 160 (80.4%) | 599 (100%) |  |
| Body mass index, Ме [Q1; Q3] | | | 30 [24; 35.1]  (n=195) | ND | - |
| Bed-day in intensive care unit (ICU),  Ме [Q1; Q3] | | | 10 (7; 14)  (n=199) | 0 | **<0.001** |
| Leukocytes, Ме [Q1; Q3] | | | 12.3 (8.9; 15.7) (n=199) | ND | - |
| Platelets, Ме [Q1; Q3] | | | 261 (193; 314) (n=199) | ND | - |
| PTI, Ме [Q1; Q3] | | | 83 [74; 92] (n=198) | ND | - |
| APTT, Ме [Q1; Q3] | | | 28.5 [25.2; 33.19] (n=199) | ND | - |
| Fibrinogen, Ме [Q1; Q3] | | | 5.12 [4.1; 6.19] (n=41) | ND | - |
| C-reactive protein (CRP),  Ме [Q1; Q3] | | | 98.5 [45.7; 146] (n=199) | ND | - |
| Groung-glass opacity, admission,  Ме [Q1; Q3] | | | 45 [20; 60] (n=199) | ND | - |
| Groung-glass opacity, discharge,  Ме [Q1; Q3] | | | 38 [10; 50] (n=187) | ND | - |
| Groung-glass opacity dynamics | | Positive. N (%) | 136 (68.34%) | ND | - |
|  |  | Negative. N (%) | 52 (26.13%) |  |  |
|  |  | ND. N (%) | 11 (5.53%) |  |  |
| Oxygen therapy day, Ме [Q1; Q3] | | | 3 [0; 5] (n=199) | - | - |
| Non-invasive mechanical ventilation,  Ме [Q1; Q3] | | | 5 [2; 8] (n=199) | - | - |
| Time to the start of clot growth  (T lag, min), Ме [Q1; Q3] | | | 1 [0.8; 1.1] (n=127) | ND | - |
| Initial spatial clot growth rates  (Vi, µm/min), Ме [Q1; Q3] | | | 39.9 [21.9; 53.3] (n=127) | ND | - |
| Stationary spatial clot growth rates  (Vs, µm/min), Ме [Q1; Q3] | | | 10.7 [6; 24.2] (n=127) | ND | - |
| Stationary spatial clot growth rates  (V, µm/min), Ме [Q1; Q3] | | | 11.5 [6.7; 25.9] (n=127) | ND | - |
| Maximum optical density of the formed clot (D), Ме [Q1; Q3] | | | 27973 [22874; 32146] (n=127) | ND | - |
| Clot size at 30 min after coagulation activation (CS, µm), Ме [Q1; Q3] | | | 670 [405; 1052] (n=127) | ND | - |
| Statistically significant differences between groups are indicated in bold; ND – no data. | | | | | |

**Supplementary Table 2.** Distribution of GWAS SNPs’ genotypes in COVID-19 hospitalized patients/healthy controls and their correspondence to the Hardy-Weinberg equilibrium

| SNP | Genotypes | Controls | *H_o_ (H_e_)*^1^ | *p*^2^ | COVID-19 patients | *H_o_* (*H_e_*)^3^ | *p*^4^ |
| --- | --- | --- | --- | --- | --- | --- | --- |
| rs143334143  *CCHCR1* | G/G | 448 (79%) | 0.1922  (0.2016) | >0.05 | 143 (77.3%) | 0.2270  (0.2013) | >0.05 |
|  | A/G | 109 (19.2%) |  |  | 42 (22.7%) |  |  |
|  | A/A | 10 (1.8%) |  |  | 0 (0%) |  |  |
|  | Maf(A) | 0.114 |  |  | 0.114 |  |  |
| rs111837807  *CCHCR1* | T/T | 453 (80%) | 0.1855  (0.1909) | >0.05 | 150 (81.1%) | 0.1892  (0.1713) | >0.05 |
|  | T/C | 105 (18.6%) |  |  | 35 (18.9%) |  |  |
|  | C/C | 8 (1.4%) |  |  | 0 (0%) |  |  |
|  | Maf(C) | 0.107 |  |  | 0.095 |  |  |
| rs17713054  *SLC6A20-LZTFL1* | A/A | 462 (81.5%) | 0.1728  (0.1780) | >0.05 | 129 (69.3%) | 0.2957  (0.2669) | >0.05 |
|  | A/G | 98 (17.3%) |  |  | 55 (29.6%) |  |  |
|  | G/G | 7 (1.2%) |  |  | 2 (1.1%) |  |  |
|  | Maf(G) | 0.099 |  |  | 0.159 |  |  |
| rs12585036  *ATP11A* | C/C | 315 (55.8%) | 0.3611  (0.3867) | >0.05 | 111 (60.3%) | 0.3533  (0.3433) | >0.05 |
|  | C/T | 204 (36.1%) |  |  | 65 (35.3%) |  |  |
|  | T/T | 46 (8.1%) |  |  | 8 (4.3%) |  |  |
|  | Maf(T) | 0.262 |  |  | 0.220 |  |  |
| rs12610495  *DPP9* | A/A | 297 (52.5%) | 0.3604  (0.4160) | <0.01 | 89 (48.6%) | 0.3825  (0.4369) | >0.05 |
|  | A/G | 204 (36%) |  |  | 70 (38.2%) |  |  |
|  | G/G | 65 (11.5%) |  |  | 24 (13.1%) |  |  |
|  | Maf(G) | 0.295 |  |  | 0.322 |  |  |
| rs17078346  *SLC6A20-LZTFL1* | A/A | 420 (74.1%) | 0.2451  (0.2360) | >0.05 | 125 (66.8%) | 0.3155  (0.2872) | >0.05 |
|  | C/A | 139 (24.5%) |  |  | 59 (31.6%) |  |  |
|  | C/C | 8 (1.4%) |  |  | 3 (1.6%) |  |  |
|  | Maf(C) | 0.137 |  |  | 0.174 |  |  |
| rs61882275  *ELF5* | G/G | 223 (39.4%) | 0.4664  (0.4676) | >0.05 | 77 (41.6%) | 0.3730  (0.4789) | <0.01 |
|  | A/G | 264 (46.6%) |  |  | 69 (37.3%) |  |  |
|  | A/A | 79 (14%) |  |  | 39 (21.1%) |  |  |
|  | Maf(A) | 0.373 |  |  | 0.397 |  |  |
| s67579710  *THBS3, THBS3-AS1* | G/G | 459 (81.2%) | 0.1788  (01772) | >0.05 | 156 (84.8%) | 0.1522  (0.1406) | >0.05 |
|  | A/G | 101 (17.9%) |  |  | 28 (15.2%) |  |  |
|  | A/A | 5 (0.9%) |  |  | 0 (0%) |  |  |
|  | Maf(A) | 0.098 |  |  | 0.076 |  |  |
| rs9636867  *IFNAR2* | A/A | 208 (36.8%) | 0.5009  (0.4719) | >0.05 | 79 (42.5%) | 0.4301  (0.4609) | >0.05 |
|  | A/G | 283 (50.1%) |  |  | 80 (43%) |  |  |
|  | G/G | 74 (13.1%) |  |  | 27 (14.5%) |  |  |
|  | Maf(G) | 0.381 |  |  | 0.36 |  |  |
| rs7949972  *ELF5* | C/C | 224 (40.1%) | 0.4606  (0.4653) | >0.05 | 79 (42.7%) | 0.4757  (0.4456) | >0.05 |
|  | C/T | 257 (46.1%) |  |  | 88 (47.6%) |  |  |
|  | T/T | 77 (13.8%) |  |  | 18 (9.7%) |  |  |
|  | Maf(T) | 0.368 |  |  | 0.335 |  |  |
| ^1^ – observed (Ho) and expected (He) heterozygosity in healthy controls; ^2^ – P-HWE in healthy controls; ^3^ – observed (Ho) and expected (He) heterozygosity in COVID-19 patients; ^4^ – P-HWE in COVID-19 patients | | | | | | | |

**Supplementary Table 3.** Results of the sex-stratified analysis of associations between GWAS SNPs and severe COVID-19 risk

| Genetic variant | Effect allele | Other allele | N | OR  [95% CI]^1^ | *p*^2^ | N | OR  [95% CI]^1^ | *p*^2^ |
| --- | --- | --- | --- | --- | --- | --- | --- | --- |
|  | |  | Males | | | Females | |  |
| rs143334143  *CCHCR1* | A | G | 292 | 0.66  [0.35-1.23] | 0.18 | 460 | 1.28  [0.81-2.02] | 0.29 |
| rs11183780  *CCHCR1* | C | T | 295 | 0.67  [0.36-1.25] | 0.19 | 456 | 1.04  [0.62-1.75] | 0.87 |
| rs17713054  *SLC6A20-LZTFL1* | A | G | 195 | **1.91**  **[1.12-3.26]** | **0.018** | 458 | **1.63**  **[1.03-2.58]** | **0.043** |
| rs17078346  *SLC6A20-LZTFL1* | C | A | 293 | 1.25  [0.75-2.08] | 0.4 | 461 | 1.44  [0.94-2.21] | 0.1 |
| rs12585036  *ATP11A* | T | C | 292 | **0.51**  **[0.32-0.83]** | **0.0039** | 457 | 1.09  [0.77-1.54] | 0.62 |
| rs12610495  *DPP9* | G | A | 292 | 0.95  [0.65-1.38] | 0.77 | 457 | 1.32  [0.96-1.80] | 0.086 |
| rs7949972  *ELF5* | T | C | 290 | 1.07  [0.74-1.57] | 0.71 | 453 | 0.75  [0.53-1.05] | 0.088 |
| rs61882275  *ELF5* | A | G | 293 | 1.32  [0.93-1.87] | 0.13 | 458 | 0.98  [0.71-1.34] | 0.88 |
| rs67579710  *THBS3, THBS3-AS1* | A | G | 292 | 0.96  [0.50-1.87] | 0.92 | 457 | 0.62  [0.34-1.15] | 0.11 |
| rs9636867  *IFNAR2* | G | A | 295 | 0.83  [0.57-1.22] | 0.34 | 456 | 0.99  [0.71-1.37] | 0.94 |
| All calculations were performed relative to the minor alleles (Effect allele); 1 - odds ratio and 95% confidence interval; 2– *p* - value; statistically significant differences are marked in bold. | | | | | | | | |

**Supplementary Table 4.** Assessment of the effect of rs17713054 SLC6A20-LZTFL1 on severe COVID-19 through comorbid diseases (mediation test results)

| Disease | Total Effect of SNP | Direct Effect of SNP | Indirect Effect of SNP |
| --- | --- | --- | --- |
| EH | -0.0639 | -0.0605 (94,68%) | -0.0035 (5,48%) |
| CAD | -0.0643 | -0.0183 (28,46%) | -0.0460 (71,54%) |
| CVA | -0.0645 | -0.0627 (97,21%) | -0.0018 (2,79%) |
| COPD | -0.0614 | -0.0500 (81.43%) | -0.0114 (18.57%) |
| T2D | -0.0632 | -0.0666 (100%) | 0.0034 (0%) |

**Supplementary Table 5.** The most significant gene-gene combinations associated with the risk of severe COVID-19 course

| Combinations of genotypes  (1) | Beta  (2) | *p*  (3) | Risk  (4) |
| --- | --- | --- | --- |
|  | | | |
| Two-locus models | | | |
| rs67579710 *THBS3, THBS3-AS1* × rs17713054 *SLC6A20-LZTFL1* |  |  |  |
| rs67579710 *THBS3, THBS3-AS1* G/G×rs17713054 *SLC6A20-LZTFL1* G/G | -0.05222 | 0.089759 | L |
| rs67579710 *THBS3, THBS3-AS1* G/G×rs17713054 *SLC6A20-LZTFL1* A/G | 0.15378 | 0.000105 | H |
| Three-locus models |  |  |  |
| rs67579710 *THBS3, THBS3-AS1* × rs17713054 *SLC6A20-LZTFL1*× rs143334143 *CCHCR1* | 0.484229 | 1.589e-05 | H |
| rs67579710 *THBS3, THBS3-AS1* G/G×rs17713054 *SLC6A20-LZTFL1* G/G×rs143334143 *CCHCR1* G/G | -0.05878 | 0.04585 | L |
| rs67579710 *THBS3, THBS3-AS1* G/G×rs17713054 *SLC6A20-LZTFL1* A/G×rs143334143 *CCHCR1* G/G | 0.16359 | 0.000228 | H |
| rs67579710 *THBS3, THBS3-AS1* G/G×rs17713054 *SLC6A20-LZTFL1* A/G×rs143334143 *CCHCR1* A/G | 0.13667 | 0.094578 | H |
| rs7949972 *ELF5* × rs67579710 *THBS3, THBS3-AS1* × rs12610495 *DPP9* |  |  |  |
| rs7949972 ELF5 T/C×rs67579710 *THBS3, THBS3-AS1* G/G×rs12610495 *DPP9* A/A | -0.06758 | 0.06965 | L |
| rs7949972 *ELF5* C/C ×rs67579710 *THBS3, THBS3-AS1* G/G×rs12610495 *DPP9* A/A | 0.09273 | 0.01719 | H |
| rs7949972 *ELF5* T/C×rs67579710 *THBS3, THBS3-AS1* A/G×rs12610495 *DPP9* A/A | 0.15491 | 0.07572 | H |
| rs7949972 *ELF5* C/C ×rs67579710 *THBS3, THBS3-AS1* A/G×rs12610495 *DPP9* A/A | -0.14452 | 0.08397 | L |
| rs7949972 *ELF5* T/C×rs67579710 *THBS3, THBS3-AS1* G/G×rs12610495 *DPP9* G/A | 0.11149 | 0.01176 | H |
| rs9636867 *IFNAR2* × rs67579710 *THBS3, THBS3-AS1* × rs17713054 *SLC6A20-LZTFL1* |  |  |  |
| rs9636867 *IFNAR2* A/G×rs67579710 *THBS3, THBS3-AS1* G/G×rs17713054 *SLC6A20-LZTFL1* G/G | -0.05784 | 0.065961 | L |
| rs9636867 *IFNAR2* G/G×rs67579710 *THBS3, THBS3-AS1* G/G×rs17713054 *SLC6A20-LZTFL1* A/G | 0.215831 | 0.000272 | H |
| rs9636867 *IFNAR2* A/A×rs67579710 *THBS3, THBS3-AS1* G/G×rs17713054 *SLC6A20-LZTFL1* A/G | 0.218347 | 0.031737 | H |
| Four-locus models |  |  |  |
| rs7949972 *ELF5* × rs9636867 *IFNAR2* × rs67579710 *THBS3, THBS3-AS1* × rs17713054 *SLC6A20-LZTFL1* |  |  |  |
| rs7949972 *ELF5* C/C ×rs9636867 *IFNAR2* G/G×rs67579710 *THBS3, THBS3-AS1* G/G×rs17713054 *SLC6A20-LZTFL1* G/G | 0.125806 | 0.015554 | H |
| rs7949972 *ELF5* C/C ×rs9636867 *IFNAR2* A/G×rs67579710 *THBS3, THBS3-AS1* G/G×rs17713054 *SLC6A20-LZTFL1* G/G | -0.09592 | 0.029776 | L |
| rs7949972 *ELF5* T/C×rs9636867 *IFNAR2* G/G×rs67579710 *THBS3, THBS3-AS1* G/G×rs17713054 *SLC6A20-LZTFL1* A/G | 0.278009 | 0.001809 | H |
| rs7949972 *ELF5* C/C ×rs9636867 *IFNAR2* G/G×rs67579710 *THBS3, THBS3-AS1* G/G×rs17713054 *SLC6A20-LZTFL1* A/G | 0.179226 | 0.044706 | H |
| rs7949972 *ELF5* T/C×rs9636867 *IFNAR2* A/G×rs67579710 *THBS3, THBS3-AS1* A/G×rs17713054 *SLC6A20-LZTFL1* A/G | 0.346928 | 0.023435 | H |
| rs7949972 *ELF5* C/C ×rs9636867 *IFNAR2* A/G×rs67579710 *THBS3, THBS3-AS1* A/G×rs17713054 *SLC6A20-LZTFL1* A/G | -0.27592 | 0.054279 | L |
| Note: obtained by the MB-MDR method, taking into account correction for covariates (gender, age);  1 – combination of genotypes;  2 – beta – logistic regression coefficients for combinations of genotypes;  3 – *p* – level of significance;  4 – Risk: H – high, L – low | | | |

**Supplementary Table 6.** Results of the analysis of associations between GWAS SNPs and severe COVID-19 risk depending on smoking status, fruit/vegetable intake, physical activity levels.

| Genetic variant | Effect allele | Other allele | N | OR  [95% CI]^1^ | *p*^2^  (*p*_bonf_) | N | OR  [95% CI]^1^ | *p*^2^  (*p*_bonf_) |
| --- | --- | --- | --- | --- | --- | --- | --- | --- |
|  |  |  | Smokers | | | Nonsmokers | | |
| rs143334143  *CCHCR1* | A | G | 216 | 1.13  [0.57-2.26] | 0.72 | 517 | 0.86  [0.55-1.36] | 0.53 |
| rs11183780  *CCHCR1* | C | T | 216 | 1.18  [0.58-2.39] | 0.65 | 516 | 0.71  [0.43-1.18] | 0.17 |
| rs17713054  *SLC6A20-LZTFL1* | A | G | 216 | 1.81  [0.87-3.78] | 0.12 | 518 | **1.65**  **[1.11-2.46]** | **0.02** |
| rs17078346  *SLC6A20-LZTFL1* | C | A | 215 | 1.65  [0.83-3.28] | 0.16 | 520 | 1.26  [0.86-1.84] | 0.24 |
| rs12585036  *ATP11A* | T | C | 215 | 0.63  [0.36-1.09] | 0.089 | 515 | 0.93  [0.67-1.28] | 0.64 |
| rs12610495  *DPP9* | G | A | 215 | 1.27  [0.82-1.98] | 0.29 | 515 | 1.07  [0.80-1.42] | 0.67 |
| rs7949972  *ELF5* | T | C | 214 | 1.01  [0.64-1.59] | 0.97 | 511 | 0.86  [0.63-1.16] | 0.31 |
| rs61882275  *ELF5* | A | G | 215 | 1.26  [0.82-1.94] | 0.28 | 517 | 1.11  [0.83-1.48] | 0.48 |
| rs67579710  *THBS3, THBS3-AS1* | A | G | 215 | 1.00  [0.40-2.49] | 0.99 | 515 | 0.73  [0.44-1.22] | 0.22 |
| rs9636867  *IFNAR2* | G | A | 216 | 1.08  [0.66-1.76] | 0.76 | 516 | 0.87  [0.65-1.17] | 0.36 |
|  |  |  | Low fruit/vegetable intake (f-) | | | Normal fruit/vegetable intake (f+) | | |
| rs143334143  *CCHCR1* | A | G | 684 | 0.97  [0.63-1.52] | 0.91  (1.82) | 629 | 0.91  [0.50-1.66] | 0.77  (1.54) |
| rs11183780  *CCHCR1* | C | T | 682 | 0.75  [0.45-1.24] | 0.24  (0.48) | 629 | 1.04  [0.58-1.87] | 0.89  (1.78) |
| rs17713054  *SLC6A20-LZTFL1* | A | G | 684 | **1.72**  **[1.15-2.58]** | **0.01**  **(0.02)** | 630 | 1.60  [0.95-2.70] | 0.088  (0.176) |
| rs17078346  *SLC6A20-LZTFL1* | C | A | 684 | 1.45  [0.99-2.14] | 0.062  (0.124) | 631 | 1.18  [0.70-1.98] | 0.54  (1.08) |
| rs12585036  *ATP11A* | T | C | 680 | 0.91  [0.66-1.26] | 0.57  (1.14) | 628 | 0.71  [0.46-1.11] | 0.12  (0.24) |
| rs12610495  *DPP9* | G | A | 681 | 1.20  [0.90-1.59] | 0.22  (0.44) | 628 | 0.98  [0.67-1.44] | 0.92  (1.84) |
| rs7949972  *ELF5* | T | C | 674 | 0.85  [0.63-1.15] | 0.29  (0.58) | 621 | 0.95  [0.65-1.40] | 0.8  (1.6) |
| rs61882275  *ELF5* | A | G | 683 | 1.16  [0.88-1.53] | 0.3  (0.6) | 628 | 1.10  [0.75-1.60] | 0.63  (1.26) |
| rs67579710  *THBS3, THBS3-AS1* | A | G | 679 | 0.60  [0.33-1.06] | 0.064  (0.128) | 629 | 1.13  [0.62-2.05] | 0.69  (1.38) |
| rs9636867  *IFNAR2* | G | A | 628 | 0.91  [0.67-1.22] | 0.51  (1.02) | 628 | 0.96  [0.65-1.42] | 0.85  (1.7) |
|  |  |  | Low physical activity level (f+) | | | Normal physical activity level (f-) | | |
| rs143334143  *CCHCR1* | A | G | 665 | 0.89  [0.54-1.45] | 0.63  (1.26) | 648 | 1.03  [0.62-1.72] | 0.9  (1.8) |
| rs11183780  *CCHCR1* | C | T | 663 | 0.65  [0.37-1.16] | 0.13  (0.26) | 648 | 1.09  [0.66-1.83] | 0.73  (1.46) |
| rs17713054  *SLC6A20-LZTFL1* | A | G | 665 | **1.93**  **[1.26-2.94]** | **0.0035**  **(0.007)** | 649 | 1.41  [0.86-2.28] | 0.18  (0.36) |
| rs17078346  *SLC6A20-LZTFL1* | C | A | 665 | 1.51  [1.00-2.28] | 0.055  (0.11) | 650 | 1.18  [0.74-1.87] | 0.49  (0.98) |
| rs12585036  *ATP11A* | T | C | 662 | 0.88  [0.62-1.25] | 0.47  (0.94) | 646 | 0.79  [0.54-1.16] | 0.22  (0.44) |
| rs12610495  *DPP9* | G | A | 662 | 1.32  [0.98-1.78] | 0.075  (0.15) | 647 | 0.90  [0.64-1.28] | 0.56  (1.12) |
| rs7949972  *ELF5* | T | C | 655 | 0.86  [0.63-1.19] | 0.37  (0.74) | 640 | 0.91  [0.65-1.29] | 0.61  (1.22) |
| rs61882275  *ELF5* | A | G | 664 | 1.11  [0.82-1.50] | 0.51  (1.02) | 647 | 1.18  [0.85-1.64] | 0.33  (0.66) |
| rs67579710  *THBS3, THBS3-AS1* | A | G | 660 | 0.96  [0.57-1.63] | 0.88  (1.76) | 648 | 0.58  [0.30-1.15] | 0.096  (0.192) |
| rs9636867  *IFNAR2* | G | A | 663 | 0.79  [0.57-1.11] | 0.17  (0.34) | 647 | 1.10  [0.78-1.54] | 0.6  (1.2) |
| All calculations were performed relative to the minor alleles (Effect allele); 1 - odds ratio and 95% confidence interval; 2– *p* - value; statistically significant differences are marked in bold. | | | | | | | | |

**Supplementary Table 7.** The most significant gene-environmental combinations associated with the risk of severe COVID-19 course

| Combinations  (1) | Beta  (2) | *p*  (3) | Risk  (4) |
| --- | --- | --- | --- |
|  | | | |
| SMOKE × rs17713054 *SLC6A20-LZTFL1* |  |  |  |
| 0×rs17713054 *SLC6A20-LZTFL1* G/G | -0.06466 | 0.03056 | L |
| 0×rs17713054 *SLC6A20-LZTFL1* A/G | 0.09025 | 0.03232 | H |
| 1×rs17713054 *SLC6A20-LZTFL1* A/G | 0.11993 | 0.06428 | H |
| SMOKE × rs9636867 *IFNAR2* |  |  |  |
| 1×rs9636867 *IFNAR2* G/G | 0.090186 | 0.05145 | H |
| 1×rs9636867 *IFNAR2* A/G | -0.08059 | 0.05344 | L |
| 1×rs9636867 *IFNAR2* A/A | 0.179684 | 0.04945 | H |
| SMOKE × rs67579710 *THBS3, THBS3-AS1* × rs17713054 *SLC6A20-LZTFL1* |  |  |  |
| 0×rs67579710 *THBS3, THBS3-AS1* G/G×rs17713054 *SLC6A20-LZTFL1* G/G | -0.04964 | 0.099296 | L |
| 0×rs67579710 *THBS3, THBS3-AS1* G/G×rs17713054 *SLC6A20-LZTFL1* A/G | 0.162513 | 0.000656 | H |
| 0×rs67579710 *THBS3, THBS3-AS1* A/G×rs17713054 *SLC6A20-LZTFL1* A/G | -0.14743 | 0.072709 | L |
| 1×rs67579710 *THBS3, THBS3-AS1* A/G×rs17713054 *SLC6A20-LZTFL1* A/G | 0.434685 | 0.031633 | H |
| SMOKE × rs9636867 *IFNAR2* × rs12585036 *ATP11A* |  |  |  |
| 1×rs9636867 *IFNAR2* G/G×rs12585036 ATP11A C/C | 0.13646 | 1.59E-02 | H |
| 1×rs9636867 *IFNAR2* A/A×rs12585036 ATP11A C/C | 0.656909 | 6.77E-05 | H |
| SMOKE × rs7949972 *ELF5* × rs9636867 *IFNAR2* × rs17713054 *SLC6A20-LZTFL1* |  |  |  |
| 0×rs7949972 *ELF5* T/C×rs9636867 *IFNAR2* G/G×rs17713054 *SLC6A20-LZTFL1* G/G | -0.0979177 | 0.060944 | L |
| 1×rs7949972 *ELF5* C/C ×rs9636867 *IFNAR2* A/G×rs17713054 *SLC6A20-LZTFL1* G/G | -0.1644537 | 0.031921 | L |
| 1×rs7949972 *ELF5* T/T×rs9636867 *IFNAR2* A/A×rs17713054 *SLC6A20-LZTFL1* G/G | 0.613844 | 0.032049 | H |
| 0×rs7949972 *ELF5* T/C×rs9636867 *IFNAR2* G/G×rs17713054 *SLC6A20-LZTFL1* A/G | 0.3137824 | 0.001539 | H |
| 1×rs7949972 *ELF5* T/C×rs9636867 *IFNAR2* A/G×rs17713054 *SLC6A20-LZTFL1* A/G | 0.3342912 | 0.004467 | H |
| 1×rs7949972 *ELF5* T/C×rs9636867 *IFNAR2* A/A×rs17713054 *SLC6A20-LZTFL1* A/G | 0.8222573 | 0.042188 | H |
| SMOKE × rs9636867 *IFNAR2* × rs12585036 *ATP11A* × rs17713054 *SLC6A20-LZTFL1* |  |  |  |
| 1×rs9636867 *IFNAR2* G/G×rs12585036 *ATP11A* C/C×rs17713054 *SLC6A20-LZTFL1* G/G | 0.151208 | 0.017234 | H |
| 1×rs9636867 *IFNAR2* A/G×rs12585036 *ATP11A* C/C×rs17713054 *SLC6A20-LZTFL1* G/G | -0.12411 | 0.041683 | L |
| 1×rs9636867 *IFNAR2* A/A×rs12585036 *ATP11A* C/C×rs17713054 *SLC6A20-LZTFL1* G/G | 0.648395 | 0.000335 | H |
| 1×rs9636867 *IFNAR2* A/A×rs12585036 *ATP11A* C/C×rs17713054 *SLC6A20-LZTFL1* A/G | 0.688554 | 0.088686 | H |
| 0×rs9636867 *IFNAR2* G/G×rs12585036 *ATP11A* C/T×rs17713054 *SLC6A20-LZTFL1* A/G | 0.331982 | 0.003276 | H |
| 0×rs9636867 *IFNAR2* A/G×rs12585036 *ATP11A* T/T×rs17713054 *SLC6A20-LZTFL1* A/G | 0.723983 | 0.073429 | H |
| 1×rs9636867 *IFNAR2* A/A×rs12585036 *ATP11A* T/T×rs17713054 *SLC6A20-LZTFL1* A/G | 0.823082 | 0.041765 | H |
| SMOKE × rs9636867 *IFNAR2* × rs12610495 *DPP9* × rs17713054 *SLC6A20-LZTFL1* |  |  |  |
| 1×rs9636867 *IFNAR2* A/G×rs12610495 *DPP9* A/A×rs17713054 *SLC6A20-LZTFL1* G/G | -0.11535 | 0.045016 | L |
| 0×rs9636867 *IFNAR2* A/A×rs12610495 *DPP9* A/A×rs17713054 *SLC6A20-LZTFL1* G/G | -0.16378 | 0.046054 | L |
| 1×rs9636867 *IFNAR2* A/A×rs12610495 *DPP9* G/A×rs17713054 *SLC6A20-LZTFL1* G/G | 0.485735 | 0.003259 | H |
| 0×rs9636867 *IFNAR2* G/G×rs12610495 *DPP9* A/A×rs17713054 *SLC6A20-LZTFL1* A/G | 0.248345 | 0.009826 | H |
| 0×rs9636867 *IFNAR2* G/G×rs12610495 *DPP9* G/A×rs17713054 *SLC6A20-LZTFL1* A/G | 0.174772 | 0.069542 | H |
| 1×rs9636867 *IFNAR2* G/G×rs12610495 *DPP9* G/A×rs17713054 *SLC6A20-LZTFL1* A/G | 0.316885 | 0.038579 | H |
| 1×rs9636867 *IFNAR2* A/G×rs12610495 *DPP9* G/G×rs17713054 *SLC6A20-LZTFL1* A/G | 0.428778 | 0.066217 | H |
| SMOKE × rs7949972 *ELF5* × rs12610495 *DPP9* × rs12585036 *ATP11A* |  |  |  |
| 1×rs7949972 *ELF5* C/C ×rs12610495 *DPP9* A/A×rs12585036 *ATP11A* C/C | 0.139597 | 0.07323 | H |
| 1×rs7949972 *ELF5* T/T×rs12610495 *DPP9* G/A×rs12585036 *ATP11A* C/C | 0.354219 | 0.05082 | H |
| 1×rs7949972 *ELF5* T/C×rs12610495 *DPP9* G/A×rs12585036 *ATP11A* C/C | 0.160743 | 0.06629 | H |
| 0×rs7949972 *ELF5* C/C ×rs12610495 *DPP9* G/A×rs12585036 *ATP11A* C/C | -0.13204 | 0.03572 | L |
| 0×rs7949972 *ELF5* C/C ×rs12610495 *DPP9* G/G×rs12585036 *ATP11A* C/C | 0.24476 | 0.03043 | H |
| 0×rs7949972 *ELF5* C/C ×rs12610495 *DPP9* A/A×rs12585036 *ATP11A* C/T | 0.157332 | 0.01429 | H |
| 1×rs7949972 *ELF5* C/C ×rs12610495 *DPP9* G/A×rs12585036 *ATP11A* C/T | -0.18251 | 0.09441 | L |
| 0×rs7949972 *ELF5* C/C ×rs12610495 *DPP9* G/A×rs12585036 *ATP11A* T/T | 0.307231 | 0.09039 | H |
| 1×rs7949972 *ELF5* C/C ×rs12610495 *DPP9* G/G×rs12585036 *ATP11A* T/T | 0.78491 | 0.05229 | H |
| Note: obtained by the MB-MDR method, taking into account correction for covariates (gender, age);  1 – combination of genotypes;  2 – beta – logistic regression coefficients for combinations of genotypes;  3 – р – level of significance;  4 – Risk: H – high, L – low;  Note: Smoking status is indicated as follows: 0 = non-smoking, 1 = smoking. | | | |

**Supplementary Table 8.** Established statistically significant associations of GWAS SNPs genotypes with clinical and biological characteristics of COVID-19 patients

| SNP | Groups | Genotypes | N | Me [Q1-Q3] | Kruskal-Wallis-Test (*p*) | Mann–Whitney  U test: *p* |
| --- | --- | --- | --- | --- | --- | --- |
| BMI | | | | | | |
| rs17713054  *SLC6A20-LZTFL1* | Entire group | G/G^1^ | 127 | 28 [23.45; 33] | **0.01** | **P^1-2^=0.0045** |
|  |  | A/G^2^ | 53 | 31.94 [27.4; 37] |  | P^1-3^=0.12 |
|  |  | A/A^3^ | 2 | 37.85 [31.4; 44.3] |  | P^2-3^=0.3 |
| rs12610495  *DPP9* | Entire group | A/A^1^ | 86 | 27.8 [23.4; 33.7] | **0.01** | P^1-2^=0.13 |
|  |  | G/A^2^ | 69 | 30 [24; 35.1] |  | **P^1-3^=0.0038** |
|  |  | G/G^3^ | 24 | 33 [28.7; 40.1] |  | P^2-3^=0.07 |
| rs17078346  *SLC6A20-LZTFL1* | Entire group | A/A^1^ | 122 | 28.5 [23.6; 33.3] | **0.01** | P^1-2^=0.06 |
|  |  | C/A^2^ | 58 | 30.8 [26; 36.9] |  | **P^1-3^=0.014** |
|  |  | C/C^3^ | 3 | 38 [37.7; 44.3] |  | **P^2-3^=0.029** |
| Oxygen therapy day | | | | | | |
| rs12610495  *DPP9* | Entire group | A/A^1^ | 89 | 4 [1; 6] | **0.02** | P^1-2^=0.483 |
|  |  | G/A^2^ | 70 | 2 [0; 7] |  | **P^1-3^=0.0155** |
|  |  | G/G^3^ | 24 | 1 [0; 3] |  | **P^2-3^=0.118** |
| The maximum optical density of the formed clot, D | | | | | | |
| rs17713054  *SLC6A20-LZTFL1* | Entire group | G/G^1^ | 79 | 26958 [22188; 31807] | **0.02** | P^1-2^=0.11 |
|  |  | A/G^2^ | 38 | 30388 [24946; 31805] |  | **P^1-3^=0.0225** |
|  |  | A/A^3^ | 2 | 35826 [35526; 36126] |  | **P^2-3^=0.0276** |
| The time of appearance of spontaneous clots Tsp, minutes | | | | | | |
| rs17713054  *SLC6A20-LZTFL1* | Entire group | G/G^1^ | 79 | 0 [0; 0] | **0.036** | P^1-2^=0.219 |
|  |  | A/G^2^ | 38 | 0 [0; 0] |  | P^1-3^=0.056 |
|  |  | A/A^3^ | 2 | 8.5 [0; 17] |  | **P^2-3^=0.004** |
| rs17713054  *SLC6A20-LZTFL1* | BM≥30 | G/G^1^ | 39 | 0 [0; 0] | **0.02** | P^1-2^=0.05 |
|  |  | A/G^2^ | 23 | 0 [0; 0] |  | P^1-3^=0.6 |
|  |  | A/A^3^ | 2 | 8.5 [0; 17] |  | **P^2-3^=0.00118** |
| rs61882275  *ELF5* | BMI<30 | G/G^1^ | 20 | 0 [0; 0] | **0.003** | P^1-2^=0.3 |
|  |  | A/G^2^ | 23 | 0 [0; 0] |  | **P^1-3^=0.031** |
|  |  | A/A^3^ | 12 | 0 [0; 16] |  | **P^2-3^=0.004** |
| The clot size at 30 min after coagulation activation, CS, μm | | | | | | |
| rs17713054  *SLC6A20-LZTFL1* | BMI<30 | G/G^1^ | 44 | 592 [344; 1013] | **0.036** | **P^1-2^=0.034** |
|  |  | A/G^2^ | 10 | 939 [703; 1175] |  | P^1-3^=1 |
|  |  | A/A^3^ | 0 | - |  | P^2-3^=1 |
| rs7949972  *ELF5* | BMI<30 | C/C^1^ | 21 | 612 [339; 808] | **0.02** | P^1-2^=0.05 |
|  |  | T/C^2^ | 30 | 737.5 [517; 1175] |  | P^1-3^=0.15 |
|  |  | T/T^3^ | 3 | 315 [0; 550] |  | **P^2-3^=0.031** |
| The time to the start of clot growth, Tlag, minutes | | | | | | |
| rs12610495  *DPP9* | BMI<30 | A/A^1^ | 30 | 1 [0.9; 1.1] | **0.01** | P^1-2^=0.24 |
|  |  | G/A^2^ | 18 | 0.9 [0.8; 1.1] |  | **P^1-3^=0.007** |
|  |  | G/G^3^ | 6 | 1.15 [1.1; 1.5] |  | **P^2-3^=0.012** |
| Stationary spatial clot growth rates Vst, μm/minutes | | | | | | |
| rs7949972  *ELF5* | BMI<30 | C/C^1^ | 21 | 10.1 [5.7; 15,8] | **0.02** | P^1-2^=0.049 |
|  |  | T/C^2^ | 30 | 11.9 [7.8; 28.8] |  | P^1-3^=0.15 |
|  |  | T/T^3^ | 3 | 3.8 [0; 10.5] |  | **P^2-3^=0.033** |

**Supplementary Table 9.** Analysis of the effect of rs17713054 *SLC6A20-LZTFL1* on the binding of DNA to transcription factors

| № | Ref/SNP allele^1^ | TF^2^ | GAIN  /LOSS^3^ | Motif^4^ | *p* -Value SNP impact^5^ | *p* -Value Ref^6^ | *p* -Value SNP^7^ |
| --- | --- | --- | --- | --- | --- | --- | --- |
| 1 | G/A | CEBPB | gain | CEBPB_2 | 0 | 0.754 | 0 |
| 2 | G/A | CEBP | gain | CEBP_5 | 0 | 0.258 | 0 |
| 3 | G/A | CEBPA | gain | CEBPA_1 | 0 | 0.794 | 0 |
| 4 | G/A | ATF1 | gain | CEBPB_2 | 0 | 0.754 | 0 |
| 5 | G/A | CEBPB | gain | CEBP_5 | 0 | 0.258 | 0 |
| 6 | G/A | EP300 | gain | CEBPA_1 | 0 | 0.794 | 0 |
| 7 | G/A | CEBPB | gain | CEBPB_1 | 0 | 0.396 | 0.000003 |
| 8 | G/A | TLX2 | gain | CEBPB_1 | 0 | 0.396 | 0.000003 |
| 9 | G/A | CEBPB | gain | CEBPB_4 | 0.0003 | 0.086 | 0.0001 |
| 10 | G/A | TEF | gain | CEBPB_4 | 0.0003 | 0.086 | 0.0001 |
| 11 | G/A | CEBPG | gain | CEBPG_3 | 0.003 | 0.091 | 0.002 |
| 12 | G/A | Ddit3::Cebpa | gain | CEBPG_3 | 0.003 | 0.091 | 0.002 |
| 13 | G/A | CEBPB | gain | CEBPB_5 | 0.002 | 0.096 | 0.003 |
| 14 | G/A | ATF4 | gain | CEBPB_5 | 0.002 | 0.096 | 0.003 |
| 15 | G/A | STAT | gain | STAT_disc4 | 0.004 | 0.075 | 0.003 |
| 16 | G/A | CEBPG | gain | CEBPG_2 | 0.003 | 0.106 | 0.004 |
| 17 | G/A | MLX | gain | CEBPG_2 | 0.003 | 0.106 | 0.004 |
| 18 | G/A | SRF | gain | SRF_3 | 0.001 | 0.061 | 0.004 |
| 19 | G/A | NFIL3 | gain | SRF_3 | 0.001 | 0.061 | 0.004 |
| 20 | G/A | CEBPB | gain | CEBPB_3 | 0.001 | 0.355 | 0.005 |
| 21 | G/A | RFX5 | gain | CEBPB_3 | 0.001 | 0.355 | 0.005 |
| 22 | G/A | CEBPE | gain | CEBPE_1 | 0.001 | 0.304 | 0.005 |
| 23 | G/A | DBP | gain | CEBPE_1 | 0.001 | 0.304 | 0.005 |
| 24 | G/A | CEBPA | gain | MA0102.3 | 0.002 | 0.110 | 0.005 |
| 25 | G/A | IRF5 | gain | MA0102.3 | 0.002 | 0.110 | 0.005 |
| 26 | G/A | CEBPA | gain | CEBPA_2 | 0.001 | 0.629 | 0.005 |
| 27 | G/A | MAFB | gain | CEBPA_2 | 0.001 | 0.629 | 0.005 |
| 28 | G/A | CEBPD | gain | CEBPD_2 | 0.001 | 0.752 | 0.006 |
| 29 | G/A | ZFX | gain | CEBPD_2 | 0.001 | 0.752 | 0.006 |
| 30 | G/A | DBP | gain | DBP_1 | 0.001 | 0.232 | 0.008 |
| 31 | G/A | CEBP | gain | DBP_1 | 0.001 | 0.232 | 0.008 |
| 32 | G/A | CEBPB | gain | MA0466.1 | 0.003 | 0.130 | 0.008 |
| 33 | G/A | RFX5 | gain | MA0466.1 | 0.003 | 0.130 | 0.008 |
| 34 | G/A | SOX5 | gain | SOX5_1 | 0.007 | 0.153 | 0.009 |
| 35 | G/A | PITX2 | gain | PITX2_1 | 0.004 | 0.200 | 0.018 |
| 36 | G/A | CUX2 | gain | CUX2_2 | 0.007 | 0.253 | 0.018 |
| 37 | G/A | CEBP | gain | CEBP_3 | 0.005 | 0.199 | 0.018 |
| 38 | G/A | DBP | gain | DBP_4 | 0.015 | 0.213 | 0.021 |
| 39 | G/A | MYC | gain | MYC_disc5 | 0.004 | 0.376 | 0.021 |
| 40 | G/A | AR | gain | MYC_disc5 | 0.004 | 0.376 | 0.021 |
| 41 | G/A | ONECUT1 | gain | ONECUT1_3 | 0.004 | 0.796 | 0.023 |
| 42 | G/A | TEF | gain | ONECUT1_3 | 0.004 | 0.796 | 0.023 |
| 43 | G/A | CUX1 | gain | CUX1_10 | 0.013 | 0.300 | 0.028 |
| 44 | G/A | HSF | gain | HSF_disc1 | 0.007 | 0.392 | 0.029 |
| 45 | G/A | ATF4 | gain | ATF4_3 | 0.006 | 0.429 | 0.035 |
| 46 | G/A | Sox5 | gain | MA0087.1 | 0.007 | 0.355 | 0.039 |
| 47 | G/A | AP1 | gain | AP1_disc1 | 0.013 | 0.440 | 0.042 |
| 48 | G/A | ONECUT2 | gain | ONECUT2_1 | 0.009 | 0.539 | 0.045 |
| 49 | G/A | NR3C1 | loss | NR3C1_2 | 0.008 | 0.004 | 0.066 |
| 50 | G/A | MSX1 | loss | MSX1_1 | 0.012 | 0.004 | 0.075 |
| 51 | G/A | MYF | loss | MYF_1 | 0.002 | 0.008 | 0.089 |
| 52 | G/A | AR | loss | MYF_1 | 0.002 | 0.008 | 0.089 |
| 53 | G/A | PAX9 | loss | PAX9_1 | 0.006 | 0.012 | 0.132 |
| 54 | G/A | RAD21 | loss | RAD21_disc8 | 0.008 | 0.031 | 0.196 |
| 55 | G/A | STAT | loss | STAT_disc7 | 0.009 | 0.028 | 0.204 |
| 56 | G/A | RUNX3 | loss | RUNX3_3 | 0.001 | 0.011 | 0.233 |
| 57 | G/A | MYC | loss | RUNX3_3 | 0.001 | 0.011 | 0.233 |
| 58 | G/A | ETS | loss | ETS_disc7 | 0.012 | 0.031 | 0.235 |
| 59 | G/A | HNF4 | loss | HNF4_disc2 | 0.004 | 0.033 | 0.267 |
| 60 | G/A | RAD21 | loss | RAD21_disc5 | 0.006 | 0.041 | 0.277 |
| 61 | G/A | RUNX2 | loss | RUNX2_5 | 0.007 | 0.017 | 0.284 |
| 62 | G/A | BCL | loss | BCL_disc9 | 0.013 | 0.048 | 0.285 |
| 63 | G/A | REST | loss | REST_disc5 | 0.009 | 0.048 | 0.285 |
| 64 | G/A | ELF1 | loss | ELF1_disc3 | 0.004 | 0.039 | 0.306 |
| 65 | G/A | MXI1 | loss | ELF1_disc3 | 0.004 | 0.039 | 0.306 |
| 66 | G/A | HINFP | loss | HINFP_4 | 0.005 | 0.034 | 0.319 |
| 67 | G/A | TFCP2 | loss | TFCP2_3 | 0.007 | 0.022 | 0.323 |
| 68 | G/A | TP73 | loss | TP73_1 | 0.015 | 0.044 | 0.331 |
| 69 | G/A | NRF1 | loss | NRF1_disc2 | 0.009 | 0.045 | 0.335 |
| 70 | G/A | E2F1 | loss | E2F1_22 | 0.007 | 0.046 | 0.475 |
| 71 | G/A | CEBP | loss | CEBP_2 | 0 | 0.172 | 0.0000004 |
| 72 | G/A | HLF | loss | CEBP_2 | 0 | 0.172 | 0.0000004 |
|  | A^8^ | integrated stress response signaling (GO:0140467; FDR = 1.48×10^-12^);  positive regulation by host of viral transcription (GO:0043923; FDR = 4.68×10^-2^);  fat cell differentiation (GO:0045444; FDR = 3.45×10^-4^);  transforming growth factor beta receptor signaling pathway (GO:0007179; FDR = 4.94×10^-2^) | | | | | |
|  | G^9^ | response to hypoxia (GO:0001666; FDR = 2.8×10^-2^) | | | | | |
| 1 – reference (Ref) / alternative (SNP) allele;  2 – TF - transcription factor;  3 – binding of TF to the reference (LOSS) / alternative (GAIN) allele;  4 – binding sites with high affinity for TF;  5 – *p* value statistically confirming the potential gain or loss of function of the genomic region with SNP in terms of transcription factor binding;  6 – *p* -value for assessing the binding of TF to the Ref allele;  7 – *p* -value for assessing the binding of TF to the SNP allele;  8 – biological processes pathogenetically significant for IS, in which TFs that bind to the SNP allele are jointly involved (data from the Gene Ontology resource; [http://geneontology.org/](http://geneontology,org/));  9 – biological processes pathogenetically significant for IS, in which TFs that bind to reference allele are jointly involved (data from the Gene Ontology resource; [http://geneontology.org/](http://geneontology,org/)) | | | | | | | |

**Supplementary Table 10.** Analysis of the effect of rs12585036 *ATP11A* on the binding of DNA to transcription factors

| № | Ref/SNP allele^1^ | TF^2^ | GAIN  /LOSS^3^ | Motif^4^ | *p* -Value SNP impact^5^ | *p* -Value Ref^6^ | *p* -Value SNP^7^ |
| --- | --- | --- | --- | --- | --- | --- | --- |
| 1 | C/T | SRY | gain | SRY_1 | 0.0004 | 0.528 | 0.001 |
| 2 | C/T | BARX1 | gain | SRY_1 | 0.0004 | 0.528 | 0.001 |
| 3 | C/T | FOXG1 | gain | SRY_1 | 0.0004 | 0.528 | 0.001 |
| 4 | C/T | FOXD1 | gain | FOXD1_2 | 0.001 | 0.109 | 0.002 |
| 5 | C/T | FOXD3 | gain | FOXD1_2 | 0.001 | 0.109 | 0.002 |
| 6 | C/T | HOXC13 | gain | FOXD1_2 | 0.001 | 0.109 | 0.002 |
| 7 | C/T | FOXD1 | gain | MA0031.1 | 0.003 | 0.052 | 0.002 |
| 8 | C/T | HOXA10 | gain | MA0031.1 | 0.003 | 0.052 | 0.002 |
| 9 | C/T | NKX3-2 | gain | MA0031.1 | 0.003 | 0.052 | 0.002 |
| 10 | C/T | FOXO1 | gain | FOXO1_1 | 0.003 | 0.098 | 0.003 |
| 11 | C/T | HOXB13 | gain | FOXO1_1 | 0.003 | 0.098 | 0.003 |
| 12 | C/T | NR3C1 | gain | FOXO1_1 | 0.003 | 0.098 | 0.003 |
| 13 | C/T | FOXJ1 | gain | FOXJ1_2 | 0.001 | 0.165 | 0.003 |
| 14 | C/T | HOXA10 | gain | FOXJ1_2 | 0.001 | 0.165 | 0.003 |
| 15 | C/T | FOXI1 | gain | FOXJ1_2 | 0.001 | 0.165 | 0.003 |
| 16 | C/T | FOXF2 | gain | FOXF2_2 | 0.0002 | 0.094 | 0.004 |
| 17 | C/T | FOXD2 | gain | FOXF2_2 | 0.0002 | 0.094 | 0.004 |
| 18 | C/T | FOXP2 | gain | FOXF2_2 | 0.0002 | 0.094 | 0.004 |
| 19 | C/T | FOXF2 | gain | MA0030.1 | 0.001 | 0.054 | 0.004 |
| 20 | C/T | HNF4 | gain | MA0030.1 | 0.001 | 0.054 | 0.004 |
| 21 | C/T | TCF7L1 | gain | MA0030.1 | 0.001 | 0.054 | 0.004 |
| 22 | C/T | HOXA11 | gain | HOXA11_3 | 0.001 | 0.294 | 0.005 |
| 23 | C/T | FOXF2 | gain | HOXA11_3 | 0.001 | 0.294 | 0.005 |
| 24 | C/T | NKX2-1 | gain | HOXA11_3 | 0.001 | 0.294 | 0.005 |
| 25 | C/T | FOXO1 | gain | FOXO1_3 | 0.007 | 0.120 | 0.005 |
| 26 | C/T | PROP1 | gain | FOXO1_3 | 0.007 | 0.120 | 0.005 |
| 27 | C/T | FOXC1 | gain | FOXC1_6 | 0.007 | 0.074 | 0.006 |
| 28 | C/T | HOXC10 | gain | FOXC1_6 | 0.007 | 0.074 | 0.006 |
| 29 | C/T | ZNF35 | gain | ZNF35_1 | 0.006 | 0.054 | 0.006 |
| 30 | C/T | FOXI1 | gain | ZNF35_1 | 0.006 | 0.054 | 0.006 |
| 31 | C/T | STAT5A | gain | STAT5A_2 | 0.001 | 0.480 | 0.006 |
| 32 | C/T | FOXO4 | gain | STAT5A_2 | 0.001 | 0.480 | 0.006 |
| 33 | C/T | TCF7L1 | gain | STAT5A_2 | 0.001 | 0.480 | 0.006 |
| 34 | C/T | HOXA13 | gain | HOXA13_2 | 0.008 | 0.152 | 0.008 |
| 35 | C/T | TCF7 | gain | HOXA13_2 | 0.008 | 0.152 | 0.008 |
| 36 | C/T | HOXD9 | gain | HOXD9_1 | 0.003 | 0.605 | 0.009 |
| 37 | C/T | PAX5 | gain | HOXD9_1 | 0.003 | 0.605 | 0.009 |
| 38 | C/T | NR2F1 | gain | HOXD9_1 | 0.003 | 0.605 | 0.009 |
| 39 | C/T | HOXA13 | gain | HOXA13_5 | 0.007 | 0.192 | 0.009 |
| 40 | C/T | NKX3-1 | gain | HOXA13_5 | 0.007 | 0.192 | 0.009 |
| 41 | C/T | FOXC1 | gain | FOXC1_3 | 0.003 | 0.138 | 0.010 |
| 42 | C/T | VENTX | gain | FOXC1_3 | 0.003 | 0.138 | 0.010 |
| 43 | C/T | HNF4G | gain | FOXC1_3 | 0.003 | 0.138 | 0.010 |
| 44 | C/T | FOXO3 | gain | FOXO3_2 | 0.002 | 0.207 | 0.011 |
| 45 | C/T | BARHL2 | gain | FOXO3_2 | 0.002 | 0.207 | 0.011 |
| 46 | C/T | HOXD12 | gain | FOXO3_2 | 0.002 | 0.207 | 0.011 |
| 47 | C/T | FOXJ3 | gain | FOXJ3_1 | 0.0004 | 0.345 | 0.013 |
| 48 | C/T | MYEF2 | gain | FOXJ3_1 | 0.0004 | 0.345 | 0.013 |
| 49 | C/T | FOXP1 | gain | FOXJ3_1 | 0.0004 | 0.345 | 0.013 |
| 50 | C/T | FOXL1 | gain | FOXL1_5 | 0.004 | 0.153 | 0.013 |
| 51 | C/T | CDX1 | gain | FOXL1_5 | 0.004 | 0.153 | 0.013 |
| 52 | C/T | HOXD9 | gain | FOXL1_5 | 0.004 | 0.153 | 0.013 |
| 53 | C/T | BARHL2 | gain | BARHL2_5 | 0.004 | 0.555 | 0.013 |
| 54 | C/T | TBP | gain | BARHL2_5 | 0.004 | 0.555 | 0.013 |
| 55 | C/T | JUN::FOS | gain | BARHL2_5 | 0.004 | 0.555 | 0.013 |
| 56 | C/T | HOXB13 | gain | HOXB13_1 | 0.006 | 0.209 | 0.014 |
| 57 | C/T | CDC5L | gain | HOXB13_1 | 0.006 | 0.209 | 0.014 |
| 58 | C/T | BARHL1 | gain | BARHL1_2 | 0.005 | 0.646 | 0.016 |
| 59 | C/T | PBX | gain | BARHL1_2 | 0.005 | 0.646 | 0.016 |
| 60 | C/T | RREB1 | gain | BARHL1_2 | 0.005 | 0.646 | 0.016 |
| 61 | C/T | HOXC11 | gain | HOXC11_3 | 0.005 | 0.316 | 0.017 |
| 62 | C/T | BSX | gain | HOXC11_3 | 0.005 | 0.316 | 0.017 |
| 63 | C/T | VDR | gain | HOXC11_3 | 0.005 | 0.316 | 0.017 |
| 64 | C/T | FOXL1 | gain | FOXL1_3 | 0.007 | 0.300 | 0.024 |
| 65 | C/T | HOXC11 | gain | FOXL1_3 | 0.007 | 0.300 | 0.024 |
| 66 | C/T | HOXC9 | gain | HOXC9_1 | 0.005 | 0.344 | 0.025 |
| 67 | C/T | FOXC1 | gain | HOXC9_1 | 0.005 | 0.344 | 0.025 |
| 68 | C/T | MNX1 | gain | HOXC9_1 | 0.005 | 0.344 | 0.025 |
| 69 | C/T | GATA4 | gain | GATA4_1 | 0.004 | 0.276 | 0.026 |
| 70 | C/T | HOXD13 | gain | GATA4_1 | 0.004 | 0.276 | 0.026 |
| 71 | C/T | FOXL1 | gain | GATA4_1 | 0.004 | 0.276 | 0.026 |
| 72 | C/T | HOXA9 | gain | HOXA9_1 | 0.005 | 0.336 | 0.028 |
| 73 | C/T | HOXD11 | gain | HOXA9_1 | 0.005 | 0.336 | 0.028 |
| 74 | C/T | AP1 | gain | HOXA9_1 | 0.005 | 0.336 | 0.028 |
| 75 | C/T | HOXD10 | gain | HOXD10_1 | 0.005 | 0.342 | 0.030 |
| 76 | C/T | FOXJ2 | gain | HOXD10_1 | 0.005 | 0.342 | 0.030 |
| 77 | C/T | NKX3-1 | gain | HOXD10_1 | 0.005 | 0.342 | 0.030 |
| 78 | C/T | PDX1 | gain | PDX1_6 | 0.007 | 0.412 | 0.031 |
| 79 | C/T | FOXK1 | gain | PDX1_6 | 0.007 | 0.412 | 0.031 |
| 80 | C/T | CDX2 | gain | MA0465.1 | 0.003 | 0.321 | 0.032 |
| 81 | C/T | AR | gain | MA0465.1 | 0.003 | 0.321 | 0.032 |
| 82 | C/T | HNF4A | gain | MA0465.1 | 0.003 | 0.321 | 0.032 |
| 83 | C/T | FOXO1 | gain | FOXO1_2 | 0.008 | 0.361 | 0.033 |
| 84 | C/T | HOXB13 | gain | FOXO1_2 | 0.008 | 0.361 | 0.033 |
| 85 | C/T | IRF | gain | IRF_2 | 0.006 | 0.373 | 0.033 |
| 86 | C/T | TATA | gain | IRF_2 | 0.006 | 0.373 | 0.033 |
| 87 | C/T | BARHL2 | gain | BARHL2_6 | 0.008 | 0.433 | 0.033 |
| 88 | C/T | UNCX | gain | BARHL2_6 | 0.008 | 0.433 | 0.033 |
| 89 | C/T | NANOG | gain | NANOG_disc4 | 0.006 | 0.364 | 0.034 |
| 90 | C/T | HOXC10 | gain | NANOG_disc4 | 0.006 | 0.364 | 0.034 |
| 91 | C/T | FOXF1 | gain | FOXF1_1 | 0.005 | 0.272 | 0.035 |
| 92 | C/T | HNF4A | gain | FOXF1_1 | 0.005 | 0.272 | 0.035 |
| 93 | C/T | NR2F1 | gain | FOXF1_1 | 0.005 | 0.272 | 0.035 |
| 94 | C/T | HOXB9 | gain | HOXB9_1 | 0.006 | 0.426 | 0.036 |
| 95 | C/T | SOX14 | gain | HOXB9_1 | 0.006 | 0.426 | 0.036 |
| 96 | C/T | PDX1 | gain | PDX1_3 | 0.008 | 0.448 | 0.037 |
| 97 | C/T | NR1H2::RXRA | gain | PDX1_3 | 0.008 | 0.448 | 0.037 |
| 98 | C/T | FOXK1 | gain | FOXK1_1 | 0.007 | 0.512 | 0.041 |
| 99 | C/T | HNF1B | gain | FOXK1_1 | 0.007 | 0.512 | 0.041 |
| 100 | C/T | MEF2A | gain | MEF2A_5 | 0.006 | 0.614 | 0.042 |
| 101 | C/T | PAX2 | gain | MEF2A_5 | 0.006 | 0.614 | 0.042 |
| 102 | C/T | FOXJ2 | gain | FOXJ2_3 | 0.002 | 0.447 | 0.048 |
| 103 | C/T | MYEF2 | gain | FOXJ2_3 | 0.002 | 0.447 | 0.048 |
| 104 | C/T | PHOX2B | gain | FOXJ2_3 | 0.002 | 0.447 | 0.048 |
| 105 | C/T | TCF7L2 | loss | TCF7L2_1 | 0.001 | 0.002 | 0.056 |
| 106 | C/T | Hoxa9 | loss | TCF7L2_1 | 0.001 | 0.002 | 0.056 |
| 107 | C/T | HOXD13 | loss | TCF7L2_1 | 0.001 | 0.002 | 0.056 |
| 108 | C/T | NR5A2 | loss | NR5A2_1 | 0 | 0 | 0.087 |
| 109 | C/T | PPARA | loss | NR5A2_1 | 0 | 0 | 0.087 |
| 110 | C/T | FOXJ3 | loss | NR5A2_1 | 0 | 0 | 0.087 |
| 111 | C/T | RXRA | loss | RXRA_3 | 0.001 | 0.010 | 0.096 |
| 112 | C/T | RFX1 | loss | RXRA_3 | 0.001 | 0.010 | 0.096 |
| 113 | C/T | IRF2 | loss | RXRA_3 | 0.001 | 0.010 | 0.096 |
| 114 | C/T | NR6A1 | loss | NR6A1_1 | 0.001 | 0.005 | 0.115 |
| 115 | C/T | PPARG | loss | NR6A1_1 | 0.001 | 0.005 | 0.115 |
| 116 | C/T | LEF1 | loss | NR6A1_1 | 0.001 | 0.005 | 0.115 |
| 117 | C/T | LEF1 | loss | LEF1_1 | 0.001 | 0.012 | 0.187 |
| 118 | C/T | TBX5 | loss | LEF1_1 | 0.001 | 0.012 | 0.187 |
| 119 | C/T | HOXD13 | loss | LEF1_1 | 0.001 | 0.012 | 0.187 |
| 120 | C/T | NR3C1 | loss | NR3C1_1 | 0.00002 | 0 | 0.220 |
| 121 | C/T | FOXQ1 | loss | NR3C1_1 | 0.00002 | 0 | 0.220 |
| 122 | C/T | TCF7L2 | loss | NR3C1_1 | 0.00002 | 0 | 0.220 |
| 123 | C/T | BDP1 | loss | BDP1_disc3 | 0.005 | 0 | 0.239 |
| 124 | C/T | Foxq1 | loss | BDP1_disc3 | 0.005 | 0.020 | 0.239 |
| 125 | C/T | ESRRA | loss | BDP1_disc3 | 0.005 | 0.020 | 0.239 |
| 126 | C/T | PGR | loss | PGR_2 | 0.003 | 0.031 | 0.393 |
| 127 | C/T | PBX1 | loss | PGR_2 | 0.003 | 0.031 | 0.393 |
| 128 | C/T | RREB1 | loss | PGR_2 | 0.003 | 0.031 | 0.393 |
| 129 | C/T | LEF1 | loss | LEF1_3 | 0.005 | 0.022 | 0.464 |
| 130 | C/T | ATF1 | loss | LEF1_3 | 0.005 | 0.022 | 0.464 |
| 131 | C/T | HOXD13 | loss | LEF1_3 | 0.005 | 0.022 | 0.464 |
| 132 | C/T | NR3C1 | loss | NR3C1_3 | 0.002 | 0.005 | 0.764 |
| 133 | C/T | FOXA2 | loss | NR3C1_3 | 0.002 | 0.005 | 0.764 |
| 134 | C/T | MYBL1 | loss | NR3C1_3 | 0.002 | 0.005 | 0.764 |
|  | T^8^ | response to testosterone (GO:0033574; FDR = 4.09×10^-11^);  androgen receptor signaling pathway (GO:0030521; FDR = 8.49×10^-3^);  canonical Wnt signaling pathway (GO:0060070; FDR = 3.54×10^-3^) | | | | | |
|  | C^9^ | - | | | | | |
| 1 – reference (Ref) / alternative (SNP) allele;  2 – TF - transcription factor;  3 – binding of TF to the reference (LOSS) / alternative (GAIN) allele;  4 – binding sites with high affinity for TF;  5 – *p* value statistically confirming the potential gain or loss of function of the genomic region with SNP in terms of transcription factor binding;  6 – *p* -value for assessing the binding of TF to the Ref allele;  7 – *p* -value for assessing the binding of TF to the SNP allele;  8 – biological processes pathogenetically significant for IS, in which TFs that bind to the SNP allele are jointly involved (data from the Gene Ontology resource; [http://geneontology.org/](http://geneontology,org/));  9 – biological processes pathogenetically significant for IS, in which TFs that bind to reference allele are jointly involved (data from the Gene Ontology resource; [http://geneontology.org/](http://geneontology,org/)) | | | | | | | |

**Supplementary Table 11.** Analysis of the effect of rs17078346 *SLC6A20-LZTFL1* on the binding of DNA to transcription factors

| № | Ref/SNP allele^1^ | TF^2^ | GAIN  /LOSS^3^ | Motif^4^ | P-Value SNP impact^5^ | P-Value Ref^6^ | P-Value SNP^7^ |
| --- | --- | --- | --- | --- | --- | --- | --- |
| 1 | A/C | T | gain | T_2 | 0.019 | 0.099 | 0.006 |
| 2 | A/C | TBX20 | gain | TBX20_3 | 0.003 | 0.132 | 0.008 |
| 3 | A/C | RFX4 | gain | TBX20_3 | 0.003 | 0.132 | 0.008 |
| 4 | A/C | T | gain | MA0009.1 | 0.001 | 0.162 | 0.012 |
| 5 | A/C | TBR1 | gain | MA0009.1 | 0.001 | 0.162 | 0.012 |
| 6 | A/C | FOXM1 | gain | FOXM1_1 | 0.005 | 0.227 | 0.015 |
| 7 | A/C | TBX21 | gain | FOXM1_1 | 0.005 | 0.227 | 0.015 |
| 8 | A/C | TBX2 | gain | TBX2_2 | 0.011 | 0.267 | 0.016 |
| 9 | A/C | MYEF2 | gain | TBX2_2 | 0.011 | 0.267 | 0.016 |
| 10 | A/C | TBX5 | gain | TBX5_3 | 0.007 | 0.190 | 0.019 |
| 11 | A/C | MYEF2 | gain | TBX5_3 | 0.007 | 0.190 | 0.019 |
| 12 | A/C | PITX1 | gain | PITX1_1 | 0.017 | 0.259 | 0.022 |
| 13 | A/C | OBOX1 | gain | OBOX1_1 | 0.002 | 0.294 | 0.023 |
| 14 | A/C | TBP | gain | OBOX1_1 | 0.002 | 0.294 | 0.023 |
| 15 | A/C | TEAD1 | gain | TEAD1_2 | 0.013 | 0.245 | 0.025 |
| 16 | A/C | NR2F2 | gain | NR2F2_1 | 0.014 | 0.186 | 0.026 |
| 17 | A/C | EOMES | gain | EOMES_1 | 0.016 | 0.233 | 0.027 |
| 18 | A/C | TBX21 | gain | TBX21_2 | 0.001 | 0.318 | 0.028 |
| 19 | A/C | ESRRG | gain | TBX21_2 | 0.001 | 0.318 | 0.028 |
| 20 | A/C | RXRA | gain | RXRA_10 | 0.003 | 0.320 | 0.028 |
| 21 | A/C | SREBP | gain | RXRA_10 | 0.003 | 0.320 | 0.028 |
| 22 | A/C | OBOX2 | gain | OBOX2_1 | 0.011 | 0.246 | 0.028 |
| 23 | A/C | MYB | gain | OBOX2_1 | 0.011 | 0.246 | 0.028 |
| 24 | A/C | MYB | gain | MYB_6 | 0.014 | 0.377 | 0.029 |
| 25 | A/C | OBOX5 | gain | OBOX5_2 | 0.010 | 0.340 | 0.032 |
| 26 | A/C | FOXA1 | gain | OBOX5_2 | 0.010 | 0.340 | 0.032 |
| 27 | A/C | SRY | gain | SRY_8 | 0.010 | 0.225 | 0.034 |
| 28 | A/C | MYBL1 | gain | SRY_8 | 0.010 | 0.225 | 0.034 |
| 29 | A/C | PBX1 | gain | PBX1_4 | 0.009 | 0.504 | 0.040 |
| 30 | A/C | Atoh1 | gain | PBX1_4 | 0.009 | 0.504 | 0.040 |
| 31 | A/C | SOX9 | gain | SOX9_7 | 0.018 | 0.217 | 0.045 |
| 32 | A/C | SIX5 | loss | SIX5_disc4 | 0.001 | 0.005 | 0.057 |
| 33 | A/C | IRX3 | loss | SIX5_disc4 | 0.001 | 0.005 | 0.057 |
| 34 | A/C | MYEF2 | loss | MYEF2_7 | 0.003 | 0.008 | 0.083 |
| 35 | A/C | FOXQ1 | loss | MYEF2_7 | 0.003 | 0.008 | 0.083 |
| 36 | A/C | HLX | loss | HLX_1 | 0.008 | 0.010 | 0.084 |
| 37 | A/C | RHOXF1 | loss | HLX_1 | 0.008 | 0.010 | 0.084 |
| 38 | A/C | IRX6 | loss | IRX6_1 | 0.010 | 0.013 | 0.124 |
| 39 | A/C | RFX3 | loss | IRX6_1 | 0.010 | 0.013 | 0.124 |
| 40 | A/C | NR3C1 | loss | MA0113.2 | 0.002 | 0.008 | 0.132 |
| 41 | A/C | CEBPB | loss | MA0113.2 | 0.002 | 0.008 | 0.132 |
| 42 | A/C | HOXD8 | loss | HOXD8_1 | 0.021 | 0.025 | 0.139 |
| 43 | A/C | IRX2 | loss | IRX2_1 | 0.016 | 0.017 | 0.143 |
| 44 | A/C | ZNF384 | loss | ZNF384_1 | 0.007 | 0.019 | 0.159 |
| 45 | A/C | MEF2A | loss | ZNF384_1 | 0.007 | 0.019 | 0.159 |
| 46 | A/C | RFX2 | loss | RFX2_3 | 0.019 | 0.029 | 0.175 |
| 47 | A/C | PROX1 | loss | PROX1_1 | 0.021 | 0.026 | 0.175 |
| 48 | A/C | RFX2 | loss | RFX2_1 | 0.022 | 0.031 | 0.203 |
| 49 | A/C | FOXO3 | loss | FOXO3_3 | 0.003 | 0.009 | 0.204 |
| 50 | A/C | SOX10 | loss | FOXO3_3 | 0.003 | 0.009 | 0.204 |
| 51 | A/C | RFX3 | loss | RFX3_2 | 0.020 | 0.032 | 0.210 |
| 52 | A/C | IRX5 | loss | IRX5_1 | 0.022 | 0.030 | 0.212 |
| 53 | A/C | TP53 | loss | TP53_4 | 0.017 | 0.038 | 0.228 |
| 54 | A/C | FOXO6 | loss | FOXO6_1 | 0.007 | 0.022 | 0.248 |
| 55 | A/C | CUX1 | loss | FOXO6_1 | 0.007 | 0.022 | 0.248 |
| 56 | A/C | CDX2 | loss | CDX2_1 | 0.001 | 0.029 | 0.269 |
| 57 | A/C | IRX3 | loss | CDX2_1 | 0.001 | 0.029 | 0.269 |
| 58 | A/C | FOXO1 | loss | FOXO1_4 | 0.002 | 0.014 | 0.275 |
| 59 | A/C | IRX4 | loss | FOXO1_4 | 0.002 | 0.014 | 0.275 |
| 60 | A/C | PAX4 | loss | PAX4_5 | 0.009 | 0.043 | 0.278 |
| 61 | A/C | Rfx1 | loss | PAX4_5 | 0.009 | 0.043 | 0.278 |
| 62 | A/C | CEBPB | loss | CEBPB_disc1 | 0.022 | 0.045 | 0.291 |
| 63 | A/C | FOXO3 | loss | FOXO3_2 | 0.013 | 0.045 | 0.325 |
| 64 | A/C | FOXD1 | loss | FOXD1_2 | 0.005 | 0.018 | 0.343 |
| 65 | A/C | CEBPB | loss | FOXD1_2 | 0.005 | 0.018 | 0.343 |
| 66 | A/C | BARHL2 | loss | BARHL2_6 | 0.015 | 0.035 | 0.349 |
| 67 | A/C | Foxq1 | loss | MA0040.1 | 0.021 | 0.043 | 0.350 |
| 68 | A/C | EP300 | loss | EP300_disc2 | 0.009 | 0.022 | 0.364 |
| 69 | A/C | CTCF | loss | EP300_disc2 | 0.009 | 0.022 | 0.364 |
| 70 | A/C | CEBPD | loss | CEBPD_2 | 0.022 | 0.045 | 0.366 |
| 71 | A/C | HOXB7 | loss | HOXB7_1 | 0.017 | 0.048 | 0.380 |
| 72 | A/C | FOXQ1 | loss | FOXQ1_1 | 0.008 | 0.042 | 0.399 |
| 73 | A/C | NR2E1 | loss | FOXQ1_1 | 0.008 | 0.042 | 0.399 |
| 74 | A/C | SRY | loss | SRY_1 | 0.013 | 0.025 | 0.530 |
| 75 | A/C | SCRT2 | loss | SCRT2_1 | 0.003 | 0.027 | 0.631 |
| 76 | A/C | CEBPA | loss | SCRT2_1 | 0.003 | 0.027 | 0.631 |
| 77 | A/C | FOXD2 | loss | FOXD2_2 | 0.015 | 0.045 | 0.751 |
|  | C^8^ | epithelial tube branching involved in lung morphogenesis (GO:0060441; FDR = 7.59×10^-4^);  vasculogenesis (GO:0001570; FDR = 7.95×10^-3^);  Notch signaling pathway (GO:0007219; FDR = 1.29×10^-3^) | | | | | |
|  | A^9^ | - | | | | | |
| 1 – reference (Ref) / alternative (SNP) allele;  2 – TF - transcription factor;  3 – binding of TF to the reference (LOSS) / alternative (GAIN) allele;  4 – binding sites with high affinity for TF;  5 – p value statistically confirming the potential gain or loss of function of the genomic region with SNP in terms of transcription factor binding;  6 – p-value for assessing the binding of TF to the Ref allele;  7 – p-value for assessing the binding of TF to the SNP allele;  8 – biological processes pathogenetically significant for IS, in which TFs that bind to the SNP allele are jointly involved (data from the Gene Ontology resource; [http://geneontology.org/](http://geneontology,org/));  9 – biological processes pathogenetically significant for IS, in which TFs that bind to reference allele are jointly involved (data from the Gene Ontology resource; [http://geneontology.org/](http://geneontology,org/)) | | | | | | | |

**Supplementary Table 12.** Analysis of the effect of rs12610495 *DPP9* on the binding of DNA to transcription factors

| № | Ref/SNP allele^1^ | TF^2^ | GAIN  /LOSS^3^ | Motif^4^ | *p* -Value SNP impact^5^ | *p* -Value Ref^6^ | *p* -Value SNP^7^ |
| --- | --- | --- | --- | --- | --- | --- | --- |
| 1 | A/G | NFE2 | gain | NFE2_disc3 | 0 | 0.228 | 3.9×10^-7^ |
| 2 | A/G | Gata4 | gain | NFE2_disc3 | 0 | 0.228 | 3.9×10^-7^ |
| 3 | A/G | E2F1 | gain | E2F1_17 | 0.002 | 0.110 | 0.002 |
| 4 | A/G | TEAD3 | gain | E2F1_17 | 0.002 | 0.110 | 0.002 |
| 5 | A/G | PITX2 | gain | PITX2_1 | 0.004 | 0.119 | 0.003 |
| 6 | A/G | HMGN3 | gain | PITX2_1 | 0.004 | 0.119 | 0.003 |
| 7 | A/G | MZF1 | gain | MZF1_3 | 0.002 | 0.125 | 0.004 |
| 8 | A/G | TAL1 | gain | MZF1_3 | 0.002 | 0.125 | 0.004 |
| 9 | A/G | MZF1_1-4 | gain | MA0056.1 | 0 | 0.128 | 0.005 |
| 10 | A/G | RFX1 | gain | MA0056.1 | 0 | 0.128 | 0.005 |
| 11 | A/G | E2F1 | gain | E2F1_16 | 0.0001 | 0.213 | 0.009 |
| 12 | A/G | ELK3 | gain | E2F1_16 | 0.0001 | 0.213 | 0.009 |
| 13 | A/G | IRF | gain | IRF_disc5 | 0.002 | 0.110 | 0.009 |
| 14 | A/G | ELK1 | gain | IRF_disc5 | 0.002 | 0.110 | 0.009 |
| 15 | A/G | E2F1 | gain | E2F1_19 | 0.001 | 0.221 | 0.011 |
| 16 | A/G | TCF12 | gain | E2F1_19 | 0.001 | 0.221 | 0.011 |
| 17 | A/G | SP1 | gain | SP1_5 | 0.001 | 0.538 | 0.012 |
| 18 | A/G | FLI1 | gain | SP1_5 | 0.001 | 0.538 | 0.012 |
| 19 | A/G | ZNF589 | gain | ZNF589_1 | 0.006 | 0.203 | 0.015 |
| 20 | A/G | ETV5 | gain | ZNF589_1 | 0.006 | 0.203 | 0.015 |
| 21 | A/G | ZIC3 | gain | ZIC3_2 | 0.004 | 0.100 | 0.015 |
| 22 | A/G | ERG | gain | ZIC3_2 | 0.004 | 0.100 | 0.015 |
| 23 | A/G | EGR1 | gain | EGR1_disc6 | 0.009 | 0.192 | 0.015 |
| 24 | A/G | ZIC2 | gain | ZIC2_2 | 0.002 | 0.135 | 0.018 |
| 25 | A/G | HDAC2 | gain | ZIC2_2 | 0.002 | 0.135 | 0.018 |
| 26 | A/G | ZIC1 | gain | ZIC1_2 | 0.004 | 0.124 | 0.019 |
| 27 | A/G | ELF3 | gain | ZIC1_2 | 0.004 | 0.124 | 0.019 |
| 28 | A/G | BCL6B | gain | BCL6B_2 | 0.004 | 0.154 | 0.019 |
| 29 | A/G | SRF | gain | BCL6B_2 | 0.004 | 0.154 | 0.019 |
| 30 | A/G | SP1 | gain | SP1_3 | 0.002 | 0.777 | 0.024 |
| 31 | A/G | EWSR1-FLI1 | gain | SP1_3 | 0.002 | 0.777 | 0.024 |
| 32 | A/G | EGR1 | gain | EGR1_disc1 | 0.003 | 0.248 | 0.025 |
| 33 | A/G | BDP1 | gain | EGR1_disc1 | 0.003 | 0.248 | 0.025 |
| 34 | A/G | USF | gain | USF_1 | 0.014 | 0.360 | 0.029 |
| 35 | A/G | ETS | gain | ETS_1 | 0.005 | 0.265 | 0.030 |
| 36 | A/G | ETV4 | gain | ETS_1 | 0.005 | 0.265 | 0.030 |
| 37 | A/G | SP1 | gain | MA0079.3 | 0.008 | 0.390 | 0.043 |
| 38 | A/G | HIC2 | gain | HIC2_1 | 0.012 | 0.884 | 0.043 |
| 39 | A/G | SP1 | gain | SP1_4 | 0.008 | 0.466 | 0.043 |
| 40 | A/G | ZBTB7A | gain | ZBTB7A_1 | 0.007 | 0.469 | 0.044 |
| 41 | A/G | ELK4 | gain | ZBTB7A_1 | 0.007 | 0.469 | 0.044 |
| 42 | A/G | MAX | gain | MAX_2 | 0.018 | 0.356 | 0.045 |
| 43 | A/G | ELF4 | loss | ELF4_1 | 0.010 | 0.003 | 0.051 |
| 44 | A/G | SPDEF | loss | SPDEF_5 | 0.008 | 0.004 | 0.069 |
| 45 | A/G | GATA3 | loss | MA0037.2 | 0.002 | 0.004 | 0.091 |
| 46 | A/G | ELK1 | loss | MA0037.2 | 0.002 | 0.004 | 0.091 |
| 47 | A/G | RUNX1 | loss | RUNX1_4 | 0.006 | 0.009 | 0.092 |
| 48 | A/G | NHLH1 | loss | RUNX1_4 | 0.006 | 0.009 | 0.092 |
| 49 | A/G | EHF | loss | EHF_2 | 0.004 | 0.004 | 0.101 |
| 50 | A/G | E2F4 | loss | EHF_2 | 0.004 | 0.004 | 0.101 |
| 51 | A/G | REST | loss | REST_disc2 | 0.015 | 0.012 | 0.104 |
| 52 | A/G | ELF1 | loss | ELF1_disc1 | 0.013 | 0.008 | 0.139 |
| 53 | A/G | Gata1 | loss | MA0035.3 | 0.002 | 0.007 | 0.180 |
| 54 | A/G | E2F1 | loss | MA0035.3 | 0.002 | 0.007 | 0.180 |
| 55 | A/G | ETS1 | loss | ETS1_4 | 0.007 | 0.008 | 0.190 |
| 56 | A/G | GATA4 | loss | ETS1_4 | 0.007 | 0.008 | 0.190 |
| 57 | A/G | GATA | loss | GATA_disc1 | 0.008 | 0.011 | 0.221 |
| 58 | A/G | Mecom | loss | GATA_disc1 | 0.008 | 0.011 | 0.221 |
| 59 | A/G | SPDEF | loss | SPDEF_6 | 0.006 | 0.008 | 0.223 |
| 60 | A/G | MZF1 | loss | SPDEF_6 | 0.006 | 0.008 | 0.223 |
| 61 | A/G | FEV | loss | MA0156.1 | 0.014 | 0.024 | 0.225 |
| 62 | A/G | ETS | loss | ETS_disc2 | 0.009 | 0.013 | 0.229 |
| 63 | A/G | GABPA | loss | MA0062.2 | 0.011 | 0.023 | 0.232 |
| 64 | A/G | GATA1 | loss | GATA1_5 | 0.013 | 0.045 | 0.247 |
| 65 | A/G | GATA1 | loss | GATA1_6 | 0.016 | 0.021 | 0.261 |
| 66 | A/G | ZBTB6 | loss | ZBTB6_1 | 0.005 | 0.012 | 0.285 |
| 67 | A/G | FEV | loss | ZBTB6_1 | 0.005 | 0.012 | 0.285 |
| 68 | A/G | ELK4 | loss | MA0076.2 | 0.003 | 0.022 | 0.288 |
| 69 | A/G | E2F1 | loss | MA0076.2 | 0.003 | 0.022 | 0.288 |
| 70 | A/G | GATA2 | loss | MA0036.2 | 0.013 | 0.028 | 0.301 |
| 71 | A/G | MEF2 | loss | MEF2_disc2 | 0.008 | 0.042 | 0.338 |
| 72 | A/G | EHF | loss | MEF2_disc2 | 0.008 | 0.042 | 0.338 |
|  | A/G | ELF1 | loss | MA0473.1 | 0.014 | 0.024 | 0.352 |
|  | A/G | TATA | loss | TATA_disc6 | 0.006 | 0.032 | 0.360 |
|  | A/G | E2F4 | loss | TATA_disc6 | 0.006 | 0.032 | 0.360 |
|  | A/G | ETS | loss | ETS_disc4 | 0.013 | 0.033 | 0.376 |
|  | A/G | GABPA | loss | GABPA_2 | 0.003 | 0.020 | 0.486 |
|  | A/G | SOX10 | loss | GABPA_2 | 0.003 | 0.020 | 0.486 |
|  | A/G | GABPA | loss | GABPA_3 | 0.001 | 0.002 | 0.629 |
|  | A/G | BCL | loss | GABPA_3 | 0.001 | 0.002 | 0.629 |
|  | A/G | CREB1 | loss | CREB1_10 | 0.011 | 0.048 | 0.633 |
|  | G^8^ | - | | | | | |
|  | A^9^ | regulation of cytokine production (GO:0001817; FDR = 0.0475) | | | | | |
| 1 – reference (Ref) / alternative (SNP) allele;  2 – TF - transcription factor;  3 – binding of TF to the reference (LOSS) / alternative (GAIN) allele;  4 – binding sites with high affinity for TF;  5 – *p* value statistically confirming the potential gain or loss of function of the genomic region with SNP in terms of transcription factor binding;  6 – *p* -value for assessing the binding of TF to the Ref allele;  7 – *p* -value for assessing the binding of TF to the SNP allele;  8 – biological processes pathogenetically significant for IS, in which TFs that bind to the SNP allele are jointly involved (data from the Gene Ontology resource; [http://geneontology.org/](http://geneontology,org/));  9 – biological processes pathogenetically significant for IS, in which TFs that bind to reference allele are jointly involved (data from the Gene Ontology resource; [http://geneontology.org/](http://geneontology,org/)) | | | | | | | |

**Supplementary Table 13.** Analysis of the effect of rs7949972 *ELF5* on the binding of DNA to transcription factors

| № | Ref/SNP allele^1^ | TF^2^ | GAIN  /LOSS^3^ | Motif^4^ | *p* -Value SNP impact^5^ | *p* -Value Ref^6^ | *p* -Value SNP^7^ |
| --- | --- | --- | --- | --- | --- | --- | --- |
| 1 | C/T | EN1 | gain | EN1_2 | 0 | 0.145 | 1.1×10^-6^ |
| 2 | C/T | NR4A2 | gain | EN1_2 | 0 | 0.145 | 1.1×10^-6^ |
| 3 | C/T | SOX14 | gain | SOX14_3 | 0.0003 | 0.068 | 0.001 |
| 4 | C/T | CREB1 | gain | SOX14_3 | 0.0003 | 0.068 | 0.001 |
| 5 | C/T | STAT | gain | STAT_disc5 | 0.0001 | 0.059 | 0.002 |
| 6 | C/T | TBX1 | gain | STAT_disc5 | 0.0001 | 0.059 | 0.002 |
| 7 | C/T | FOXA | gain | FOXA_disc4 | 0.002 | 0.053 | 0.002 |
| 8 | C/T | AR | gain | FOXA_disc4 | 0.002 | 0.053 | 0.002 |
| 9 | C/T | SPI1 | gain | SPI1_disc1 | 0.004 | 0.062 | 0.004 |
| 10 | C/T | SPIB | gain | SPIB_2 | 0.002 | 0.104 | 0.005 |
| 11 | C/T | TFCP2 | gain | SPIB_2 | 0.002 | 0.104 | 0.005 |
| 12 | C/T | SOX2 | gain | SOX2_3 | 0.024 | 0.052 | 0.009 |
| 13 | C/T | SOX1 | gain | SOX1_3 | 0.003 | 0.130 | 0.010 |
| 14 | C/T | NR6A1 | gain | SOX1_3 | 0.003 | 0.130 | 0.010 |
| 15 | C/T | ESRRB | gain | ESRRB_2 | 0.024 | 0.071 | 0.010 |
| 16 | C/T | NR5A1 | gain | NR5A1_2 | 0.009 | 0.170 | 0.010 |
| 17 | C/T | SOX8 | gain | SOX8_6 | 0.009 | 0.145 | 0.012 |
| 18 | C/T | SOX8 | gain | SOX8_5 | 0.023 | 0.060 | 0.012 |
| 19 | C/T | Esrrb | gain | MA0141.2 | 0.005 | 0.144 | 0.012 |
| 20 | C/T | ESRRB | gain | ESRRB_1 | 0.013 | 0.149 | 0.012 |
| 21 | C/T | ESRRG | gain | ESRRG_2 | 0.017 | 0.122 | 0.014 |
| 22 | C/T | EP300 | gain | EP300_disc2 | 0.004 | 0.430 | 0.014 |
| 23 | C/T | PBX | gain | PBX_1 | 0.006 | 0.220 | 0.016 |
| 24 | C/T | EGR1 | gain | EGR1_disc4 | 0.005 | 0.161 | 0.017 |
| 25 | C/T | SPI1 | gain | SPI1_3 | 0.010 | 0.348 | 0.022 |
| 26 | C/T | ELF1 | gain | ELF1_1 | 0.008 | 0.296 | 0.022 |
| 27 | C/T | HNF4A | gain | HNF4A_7 | 0.025 | 0.142 | 0.023 |
| 28 | C/T | ZNF652 | gain | ZNF652_1 | 0.003 | 0.567 | 0.024 |
| 29 | C/T | CEBPB | gain | MA0466.1 | 0.014 | 0.258 | 0.026 |
| 30 | C/T | ESRRA | gain | ESRRA_4 | 0.013 | 0.216 | 0.028 |
| 31 | C/T | ESRRG | gain | ESRRG_3 | 0.023 | 0.290 | 0.029 |
| 32 | C/T | CREB3 | loss | CREB3_2 | 0.024 | 0.242 | 0.029 |
| 33 | C/T | STAT6 | loss | STAT6_2 | 0.009 | 0.316 | 0.034 |
| 34 | C/T | SOX10 | loss | SOX10_5 | 0.020 | 0.180 | 0.037 |
| 35 | C/T | SIX5 | loss | SIX5_disc3 | 0.019 | 0.178 | 0.043 |
| 36 | C/T | EWSR1::FLI1 | loss | EWSR1::FLI1_1 | 0.001 | 0.312 | 0.046 |
| 37 | C/T | NR2F6 | loss | EWSR1::FLI1_1 | 0.001 | 0.312 | 0.046 |
| 38 | C/T | POU3F2 | loss | POU3F2_2 | 0.024 | 0.211 | 0.048 |
| 39 | C/T | IRF2 | loss | IRF2_1 | 0.0001 | 0.003 | 0.055 |
| 40 | C/T | REST | loss | IRF2_1 | 0.0001 | 0.003 | 0.055 |
| 41 | C/T | ZBTB33 | loss | ZBTB33_disc4 | 0.001 | 0.002 | 0.058 |
| 42 | C/T | ESRRA | loss | ZBTB33_disc4 | 0.001 | 0.002 | 0.058 |
| 43 | C/T | CREB1 | loss | CREB1_2 | 0.001 | 0.002 | 0.084 |
| 44 | C/T | IRF5 | loss | CREB1_2 | 0.001 | 0.002 | 0.084 |
| 45 | C/T | STAT | loss | STAT_disc3 | 0.016 | 0.022 | 0.141 |
| 46 | C/T | TEAD2 | loss | TEAD2_1 | 0.0007 | 0.008 | 0.170 |
| 47 | C/T | RXRA | loss | TEAD2_1 | 0.0007 | 0.008 | 0.170 |
| 48 | C/T | IRF1 | loss | IRF1_1 | 0.014 | 0.016 | 0.180 |
| 49 | C/T | IRF9 | loss | IRF9_1 | 0.011 | 0.017 | 0.203 |
| 50 | C/T | IRF9 | loss | IRF9_2 | 0.010 | 0.017 | 0.227 |
| 51 | C/T | CREB1 | loss | CREB1_8 | 0.021 | 0.019 | 0.250 |
| 52 | C/T | PATZ1 | loss | PATZ1_1 | 0.0002 | 0.025 | 0.271 |
| 53 | C/T | CEBPA | loss | PATZ1_1 | 0.0002 | 0.025 | 0.271 |
| 54 | C/T | ZNF740 | loss | ZNF740_3 | 0.014 | 0.046 | 0.326 |
| 55 | C/T | IRF3 | loss | IRF3_1 | 0.022 | 0.048 | 0.333 |
| 56 | C/T | RUNX2 | loss | RUNX2_6 | 0.003 | 0.016 | 0.341 |
| 57 | C/T | RUNX2 | loss | RUNX2_1 | 0.002 | 0.005 | 0.381 |
| 58 | C/T | NR2F1 | loss | RUNX2_1 | 0.002 | 0.005 | 0.381 |
| 59 | C/T | RUNX1 | loss | RUNX1_7 | 0.013 | 0.035 | 0.382 |
| 60 | C/T | Gfi1b | loss | MA0483.1 | 0.025 | 0.044 | 0.391 |
| 61 | C/T | REST | loss | REST_disc4 | 0.007 | 0.045 | 0.624 |
| 62 | C/T | RUNX3 | loss | RUNX3_2 | 0.004 | 0.021 | 0.813 |
| 63 | C/T | OVOL2 | loss | OVOL2_1 | 0.009 | 0.048 | 0.865 |
|  | T^8^ | - | | | | | |
|  | C^9^ | positive regulation of CD8-positive, alpha-beta T cell differentiation (GO:0043378; FDR = 0.00247);  negative regulation of CD4-positive, alpha-beta T cell differentiation (GO:0043371; FDR = 0.0301);  defense response to virus (GO:0051607; FDR = 0.00177)  positive regulation of interferon-alpha production (GO:0032727; FDR = 0.0413);  positive regulation of interferon-beta production (GO:0032728; 0.00251) | | | | | |
| 1 – reference (Ref) / alternative (SNP) allele;  2 – TF - transcription factor;  3 – binding of TF to the reference (LOSS) / alternative (GAIN) allele;  4 – binding sites with high affinity for TF;  5 – *p* value statistically confirming the potential gain or loss of function of the genomic region with SNP in terms of transcription factor binding;  6 – *p* -value for assessing the binding of TF to the Ref allele;  7 – *p* -value for assessing the binding of TF to the SNP allele;  8 – biological processes pathogenetically significant for IS, in which TFs that bind to the SNP allele are jointly involved (data from the Gene Ontology resource; [http://geneontology.org/](http://geneontology,org/));  9 – biological processes pathogenetically significant for IS, in which TFs that bind to reference allele are jointly involved (data from the Gene Ontology resource; [http://geneontology.org/](http://geneontology,org/)) | | | | | | | |
